# Comparative Analysis of GC-MS Chemical Profiles and Multidimensional Biological Activities of Essential Oils from Thirteen *Lamiaceae* Plants

**DOI:** 10.3390/plants15203084

**Published:** 2026-10-09

**Authors:** Feng Han, Yingmei Ma, Yuting Bai, Shuangyi Zhang, Yan Zhao, Xiujie Yin

**Affiliations:** 1 College of Grassland Science, Inner Mongolia Agricultural University, Hohhot 010011, China; hfnmd@imau.edu.cn; 2 Key Laboratory of National Forestry and Grassland Administration on Native Grass Breeding, College of Grassland Science, Inner Mongolia Agricultural University, Hohhot 010018, China; 3 College of Desert Management, Inner Mongolia Agricultural University, Hohhot 010018, China; mym_youxiang@sina.com; 4 College of Veterinary Medicine, Inner Mongolia Agricultural University, Hohhot 010011, China; hastinhim@imau.edu.cn (Y.B.); zhangshuangyi@imau.edu.cn (S.Z.); 5 College of Animal Science and Technology, Northeast Agricultural University, Harbin 150030, China

**Keywords:** *Lamiaceae*, essential oil, chemical composition, antioxidant, antibacterial, anti-inflammatory, cytotoxicity

## Abstract

*Lamiaceae* plants produce essential oils (EOs) with diverse chemical compositions and biological activities. This study investigated the volatile composition and multiple biological activities of EOs from 13 *Lamiaceae* species. EOs were obtained by hydrodistillation and characterized by gas chromatography–mass spectrometry (GC-MS), followed by hierarchical clustering and principal component analysis. Antioxidant, antibacterial, cytotoxic, and anti-inflammatory activities were evaluated using in vitro and murine models. GC-MS analysis revealed substantial interspecific variation in volatile composition, with *Origanum vulgare* characterized by carvacrol and limonene, *Thymus* spp. by thymol, *Rosmarinus officinalis* by eucalyptol and α-pinene, *Mentha canadensis* by L-menthol, and *Perilla frutescens* by perillaldehyde. *Scutellaria baicalensis*, *Elsholtzia ciliata*, *Phlomis mongolica*, and *Schizonepeta tenuifolia* showed distinctive profiles associated with limonene and isopulegone, nepetalactone, pulegone, and anethole and anisaldehyde, respectively. Multivariate analysis further separated the 13 oils into three chemotype groups: a carvacrol-rich *Origanum* group, a *Scutellaria* and *Thymus* group enriched in sesquiterpenes or thymol, and a group comprising the remaining six genera dominated by shared monoterpene hydrocarbons. Most EOs exhibited substantial 2,2-diphenyl-1-picrylhydrazyl (DPPH) radical-scavenging activity, with *Rosmarinus officinalis* and *Perilla frutescens* showing the strongest activity, with IC_50_ values of 2.14 and 2.08 μg/mL, respectively. *Origanum vulgare*, *Thymus citriodorus*, *Thymus vulgaris*, and *Thymus mongolicus* displayed strong antibacterial activity against five pathogens, with minimum inhibitory concentration (MIC) values ranging from 2 to 8 μg/mL. Cytotoxicity assays showed potent activity against HepG2 and LNCaP cells, with IC_50_ values ranging from 0.31 to 0.95 μg/mL. *Elsholtzia ciliata* and *Perilla frutescens* exhibited particularly strong potency, with IC_50_ values ranging from 0.31 to 0.40 μg/mL. In a murine skin inflammation model, selected EOs significantly reduced inflammation-related markers, including COX-2, TNF-α, NF-κB, IKK, Akt, and PKC. Correlation analysis identified significant associations of carvacrol, thymol, and β-linalool with antibacterial activity, whereas no significant associations were observed between individual major constituents and antioxidant or cytotoxic activity after false discovery rate (FDR) correction. Overall, the 13 *Lamiaceae* EOs displayed distinct chemical profiles and activity patterns, providing a basis for further investigation of their bioactive constituents and potential applications.

## 1. Introduction

The *Lamiaceae* family consists of approximately 200 genera and over 3500 species and is widely distributed across the world. In China, there are 102 genera and approximately 800 species of *Lamiaceae*, including *Mentha*, *Scutellaria*, *Origanum*, *Nepeta*, *Elsholtzia*, and *Thymus* [1]. The majority of *Lamiaceae* plants are rich in aromatic and non-volatile compounds and have considerable medicinal and functional value [2]. The stems and leaves of most Lamiaceae plants are covered with glandular trichomes, which are the primary sites for the synthesis, accumulation, and storage of essential oils [3]. The pharmacological significance of *Lamiaceae* volatile oils has been well-documented across multiple genera. For instance, the essential oil (EO) of *Schizonepeta* spp. has been demonstrated to exhibit a broad spectrum of pharmacological activities, including anti-inflammatory, antioxidant, antimicrobial, antiviral, insecticidal, hemostatic, and antitumor effects [4,5]. Similarly, EOs derived from *Agastache* spp. have shown remarkable antibacterial and cytotoxic activities, displaying potent inhibitory effects against pathogenic bacteria such as *Escherichia coli* (*E. coli*) and *Staphylococcus aureus* (*S. aureus*), as well as significant cytotoxicity against triple-negative breast cancer and hepatocellular carcinoma cell lines [6]. Furthermore, *Perilla frutescens*, a traditional medicinal and edible plant widely cultivated in East Asia, has been reported to possess multiple therapeutic properties, including anti-inflammatory, antimicrobial, antioxidant, and hepatoprotective activities [7].

Microbial contamination of food products poses a persistent challenge to the food industry and public health. In this context, plant-derived EOs have attracted considerable attention as natural flavoring, preservative, and antimicrobial agents. Several *Lamiaceae* EOs, including oregano and peppermint oils, have demonstrated antibacterial activity against foodborne pathogens such as *E. coli* and *S. aureus*, supporting their potential applications in food preservation [8,9,10]. Beyond their antimicrobial applications, *Lamiaceae* EOs have also attracted considerable interest for their potential anticancer and anti-inflammatory activities. Previous studies have reported that EOs from species such as *Origanum vulgare* and *Rosmarinus officinalis* can inhibit tumor growth or cancer-related cellular processes, with effects associated with apoptosis and modulation of relevant signaling pathways [11,12,13,14]. These findings further highlight the diverse biological activities of *Lamiaceae* EOs and support their continued investigation as potential sources of bioactive compounds.

Previous comparative studies have investigated the chemical composition and selected biological activities of EOs from multiple *Lamiaceae* species [15,16]. However, differences in plant materials, geographical origin, harvesting conditions, developmental stage, EO extraction procedures, analytical platforms, and bioactivity assays may limit direct comparisons across species and studies. Moreover, previous comparative investigations have generally focused on selected biological endpoints, whereas comparative assessments integrating chemical profiling with multiple biological activities under a relatively consistent experimental framework remain limited. To address this gap, the present study selected 13 *Lamiaceae* species and systematically characterized their EO compositions and antioxidant, antibacterial, cytotoxic, and anti-inflammatory activities using a standardized experimental workflow. Multivariate analysis was used to characterize differences in EO chemical composition, while correlation analysis was performed as an exploratory approach to examine potential associations between major volatile constituents and biological activities. These associations were interpreted as exploratory and were not used to infer direct causality or experimentally validated synergistic interactions. By integrating chemical composition profiles with multiple biological endpoints, this study aimed to comparatively characterize the chemical diversity and multidimensional bioactivity patterns of 13 *Lamiaceae* EOs and to provide testable hypotheses for subsequent mechanistic investigations.

## 2. Results

### 2.1. Plant Resources and EO Extraction

*Lamiaceae* plants are widely distributed across almost all regions of China. China has approximately 99 genera and over 800 species of *Lamiaceae* plants, accounting for about 25% of the global total. The color of EOs extracted from these plants can be light yellow, yellow, or orange-red. Representative photographs of the 13 extracted EOs showing their characteristic color differences are provided in Supplementary Figure S1. In this study, the yield of EO (wt %) from 13 *Lamiaceae* plants ranged from 1.47% to 3.16%. The highest yield was from *Origanum vulgare* (3.16%), followed by *Nepeta cataria* (3.12%), *Elsholtzia ciliata* (2.84%), *Perilla frutescens* (2.16%), *Mentha canadensis* (2.13%), *Scutellaria baicalensis* (1.83%), *Rosmarinus officinalis* (1.79%), *Schizonepeta tenuifolia* (1.73%), and *Scutellaria scordifolia* (1.52%) [17]. *Phlomis mongolica* exhibited the lowest EO yield (1.47%), followed by species of the *Thymus* genus (*Thymus citriodorus*, *Thymus vulgaris*, and *Thymus mongolicus*; 1.50%) (Table 1).

### 2.2. GC-MS Analysis

The total ion current chromatograms (TIC) of 13 EOs were obtained using gas chromatography–mass spectrometry (GC-MS), as shown in Figure S2. The TIC profiles comprehensively demonstrated the composition of volatile compounds in each EO, revealing significant differences in the number of chromatographic peaks, retention time, and relative abundance among different species. The mass spectra of the main chromatographic peaks were compared with the NIST mass spectrometry database of the United States National Institute of Standards and Technology (NIST). Experimental retention indices (RI exp) were calculated using n-alkane standards (C_6_–C_40_) and compared with retention indices reported in the NIST database and published literature (RI lit) to assist compound identification. Based on this, a systematic analysis of the chemical composition of 13 EOs from the *Lamiaceae* family was conducted, and a total of 51 volatile compounds were identified (Table 2), mainly including monoterpenes, sesquiterpenes, and oxygen-containing derivatives. The main volatile compounds with a relative content exceeding 5% in each EO are summarized in Table S1, and the distribution of major components exhibited significant interspecific differences and chemical diversity among the species.

*Rosmarinus officinalis* was characterized by high relative contents of α-pinene (16.00%) and eucalyptol (38.00%); *Scutellaria baicalensis* and *Origanum vulgare* were rich in limonene (41.78% and 34.48%, respectively); *Nepeta cataria* and *Elsholtzia ciliata* contained high proportions of iridoid nepetalactone (16.30% and 35.00%, respectively); *Mentha canadensis* had an extremely high content of L-menthol (34.83%); *Perilla frutescens* had a high content of perilla aldehyde (35.00%); *Origanum vulgare* was rich in carvacrol (28.00%); *Scutellaria baicalensis* contained a large amount of isopulegone (10.94%); and samples from the *Thymus* genus (*Thymus vulgaris*, *Thymus mongolicus*, and *Thymus citriodorus*) were mainly characterized by high contents of thymol (11.00%, 13.30%, and 25.00%, respectively). With regard to the abundance of specific compounds, limonene exhibited the highest relative content in *Scutellaria baicalensis* (41.78%), whereas (Z)-nerolidol and bornylene were present at very low relative abundances in most samples, representing trace components [16]. The above analysis revealed significant differences in the chemical fingerprint profiles of the EOs from different genera, reflecting the diversity of their volatile chemical compositions.

### 2.3. Chemical Profiling and Multivariate Analysis

**Figure 1:**
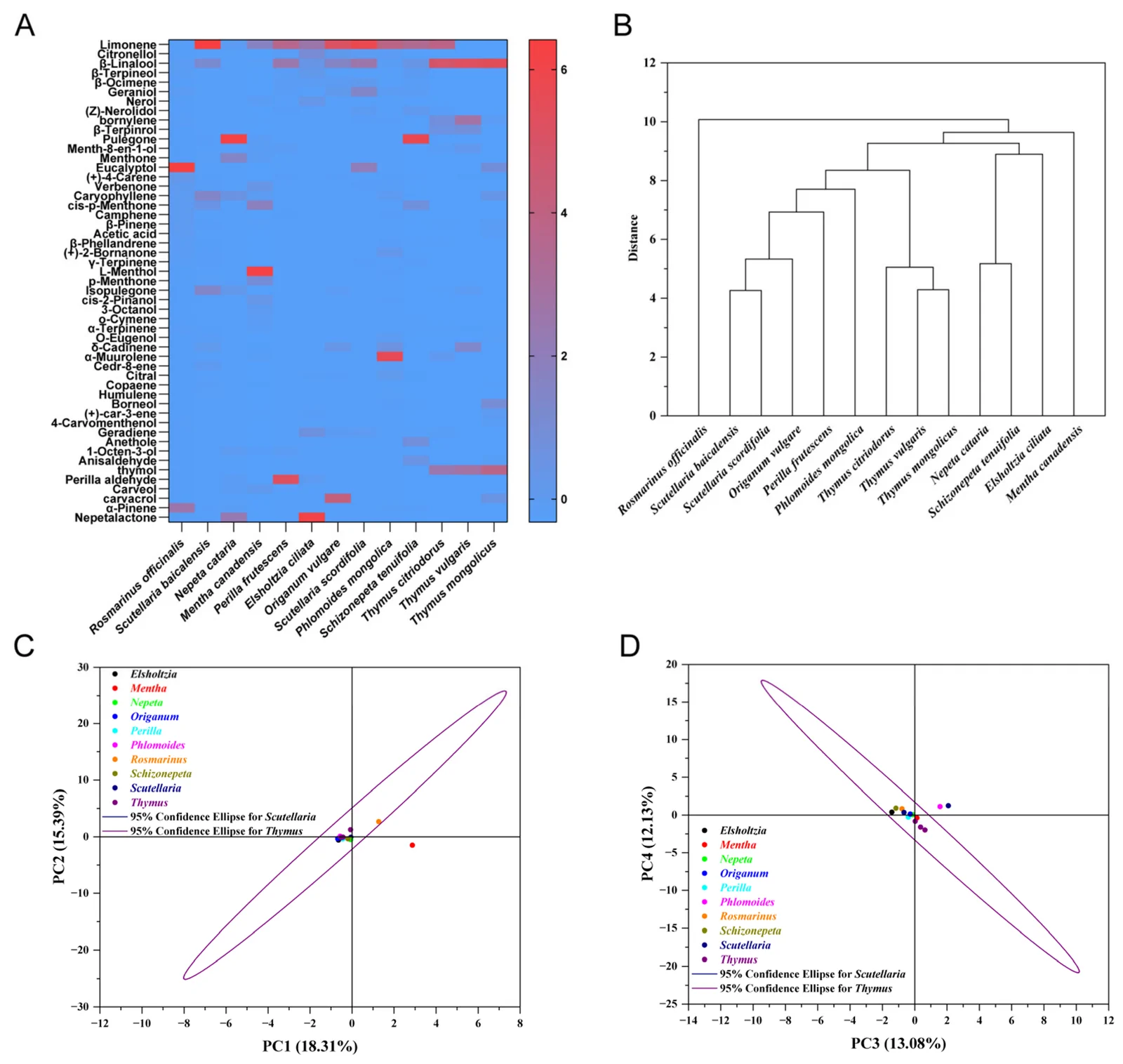
GC-MS volatile profiles of 13 *Lamiaceae* EOs. (**A**) Heatmap of z-score-normalized compound abundances. (**B**) Corresponding hierarchical clustering dendrogram generated using Euclidean distance and Ward’s linkage method. (**C**) PCA score plot of PC1 versus PC2 and (**D**) PCA score plot of PC3 versus PC4; the percentage of variance explained by each principal component is indicated on the corresponding axis. Colors denote different *Lamiaceae* genera, as indicated in the plot legends.

To comprehensively visualize the chemical relationships among the 13 EOs, hierarchical clustering analysis (HCA) and principal component analysis (PCA) were performed based on the GC-MS volatile profiles (Figure 1).

A hierarchical clustering heatmap (Figure 1A) and the corresponding dendrogram (Figure 1B) were constructed to reveal the similarity patterns of major volatile compounds across samples. In the heatmap, the color scale from blue to red represents row-wise z-score-standardized compound abundances, indicating relative enrichment (red) or depletion (blue) across samples. Both samples and compounds were clustered using Euclidean distance and Ward’s linkage method. At the compound level, based on their distribution patterns and structural features, three compound clusters (A–C) were distinguished. Cluster A (phenolic monoterpenes: carvacrol and thymol) was highly accumulated in *Origanum* and *Thymus* (red) but nearly undetectable in other genera (dark blue), representing characteristic compounds of these genera. Cluster B (sesquiterpenes: β-caryophyllene, caryophyllene oxide, and α-muurolene) was enriched in specific genera, e.g., *Scutellaria scordifolia* with 31.93% α-muurolene. Cluster C (monoterpene hydrocarbons: α-pinene, limonene, and p-menthene) was ubiquitously distributed across most genera as shared skeletal components.

At the sample level, the 13 oils were classified into three chemotype groups (I–III). Group I comprised only *Origanum*, characterized by extreme accumulation of carvacrol (the dominant chemotaxonomic marker), accompanied by relatively high levels of limonene as a minor co-occurring monoterpene hydrocarbon. Group II included *Scutellaria* and *Thymus* as two sub-clusters: *Scutellaria* was enriched in sesquiterpenes (Cluster B), whereas *Thymus* was dominated by moderate-to-high levels of both monoterpene hydrocarbons (Cluster C) and phenolic monoterpenes (Cluster A). Group III contained the remaining six genera (*Elsholtzia*, *Mentha*, *Nepeta*, *Perilla*, *Phlomis*, and *Schizonepeta*), which exhibited moderately similar color patterns dominated by common monoterpene hydrocarbons with no genus-specific markers; however, *Phlomis mongolica* (40.00% pulegone) and *Schizonepeta tenuifolia* (characterized by phenylpropanoids anethole and anisaldehyde) showed distinct chemical features, indicating some degree of heterogeneity within this group.

PCA was performed to complement the clustering results and to explore the multidimensional variance structure of the dataset (Figure 1C,D). The first four principal components (PC1–PC4) collectively accounted for 58.88% of the total variance. In the PC1–PC2 score plot (Figure 1C), *Mentha* and *Rosmarinus* were clearly separated from the other genera along the positive directions of PC1 and PC2, respectively, which was associated with the high contents of L-menthol (34.83%) and α-pinene (16.00%)/eucalyptol (38.00%). Meanwhile, the remaining genera, including *Origanum*, *Thymus*, and *Scutellaria*, were positioned near the origin, indicating that the primary variance captured by PC1 and PC2 was predominantly driven by the distinct chemical profiles of *Mentha* and *Rosmarinus*. Among the genera distributed around the origin, *Phlomis* (40.00% pulegone) and *Schizonepeta* (characterized by anethole and anisaldehyde) exhibited moderate deviations, consistent with their distinctive chemical features observed in the heatmap.

In the PC3–PC4 score plot (Figure 1D), the separation pattern changed markedly, revealing finer chemical variations that were not captured by the first two principal components. *Scutellaria* was positioned in the positive direction of PC3, which may be attributed to its limonene-rich profile (41.78%). *Thymus* was positioned in the negative direction of PC4, possibly due to its enrichment in thymol and other oxygenated monoterpenes. These higher-order separation patterns were broadly consistent with the sub-clustering patterns observed in the heatmap and dendrogram, indicating that PC3 and PC4 captured additional chemical variation that was not apparent in the PC1–PC2 space.

Taken together, the hierarchical clustering and PCA analyses demonstrated substantial interspecific differences in the volatile chemical composition of the 13 *Lamiaceae* EOs and identified several characteristic chemotypes. *Origanum vulgare* was distinguished by a carvacrol-rich profile, whereas the *Thymus* oils were characterized by relatively high levels of thymol. *Rosmarinus officinalis*, *Perilla frutescens*, and *Mentha canadensis* showed distinctive oxygenated-monoterpene or monoterpenoid profiles dominated by eucalyptol, perillaldehyde, and L-menthol, respectively. *Scutellaria baicalensis* was characterized by a limonene-rich profile, while *Elsholtzia ciliata* and *Nepeta cataria* showed relatively high proportions of nepetalactone. In addition, *Phlomis mongolica* and *Schizonepeta tenuifolia* displayed distinctive pulegone- and phenylpropanoid-rich profiles, respectively.

### 2.4. Antioxidant Activity

**Figure 2:**
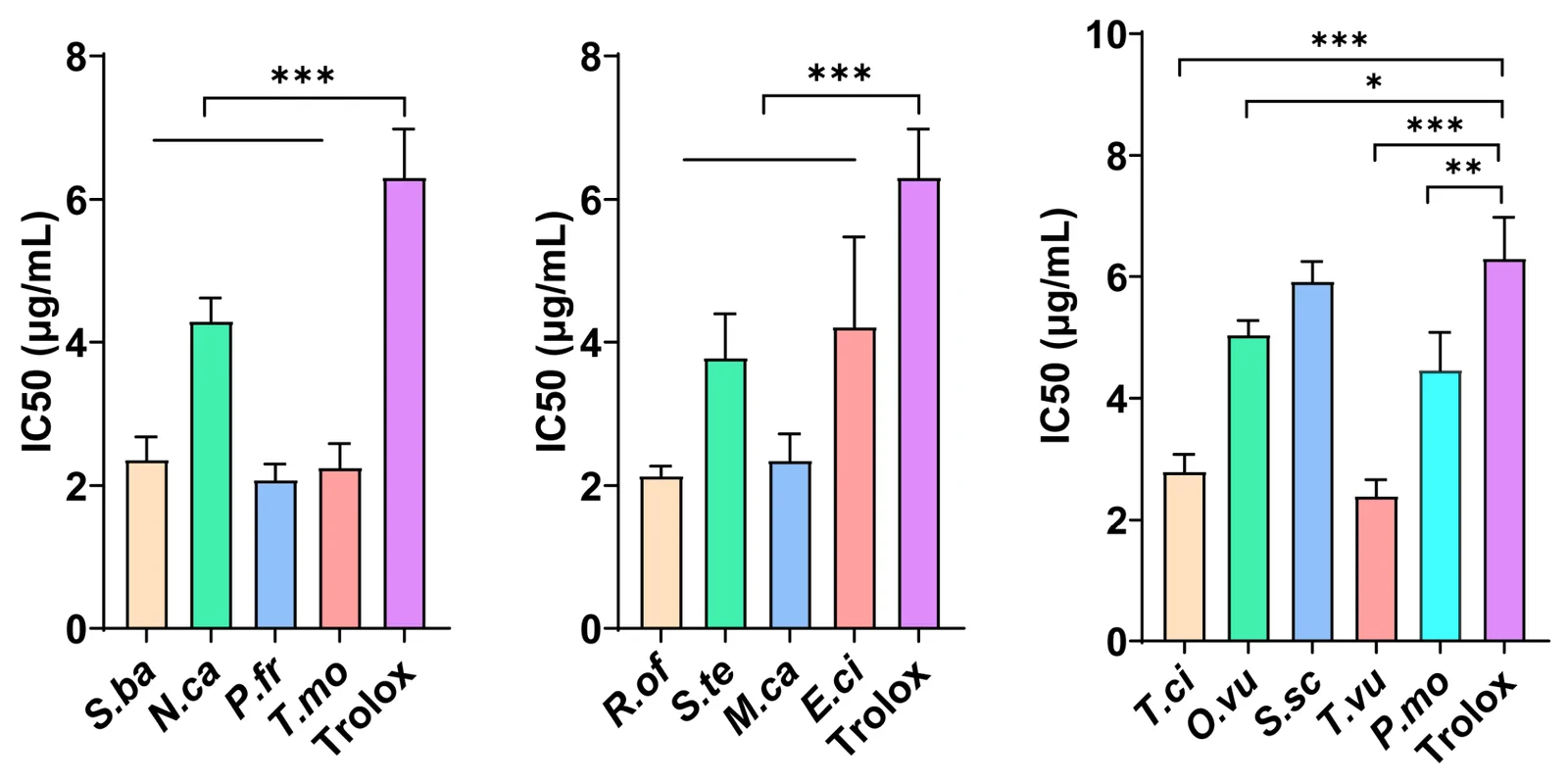
IC_50_ of 13 *Lamiaceae* EOs of DPPH. Values are presented as mean ± SD from three independent experiments. Asterisks indicate statistically significant differences compared with the control: ^*^ *p* < 0.05, ^**^p < 0.01, ^***^p < 0.001.

The antioxidant activities of the 13 EOs were evaluated using the 2,2-Diphenyl-1-picrylhydrazyl (DPPH) radical-scavenging assay. The IC_50_ values are presented in Figure 2, while the single-concentration DPPH scavenging rates are provided in Table S2. The IC_50_ values ranged from 2.08 to 5.92 μg/mL. *Perilla frutescens* (2.08 μg/mL) and *Rosmarinus officinalis* (2.14 μg/mL) exhibited the strongest antioxidant activities, followed by *Thymus mongolicus* (2.25 μg/mL), *Mentha canadensis* (2.35 μg/mL), and *Scutellaria baicalensis* (2.36 μg/mL). The positive control Trolox showed an IC_50_ value of 6.31 μg/mL under the same assay conditions. The single-concentration DPPH scavenging rates showed a generally consistent pattern.

The differences in antioxidant activity were observed across EOs with distinct chemical profiles. *Rosmarinus officinalis* was characterized by a high relative abundance of eucalyptol (38.00%), *Perilla frutescens* by perillaldehyde (35.00%), *Scutellaria baicalensis* by limonene (41.78%), *Mentha canadensis* by L-menthol (34.83%), and the *Thymus* oils by relatively high levels of thymol. However, Spearman’s rank correlation analysis after Benjamini–Hochberg false discovery rate (FDR) correction revealed no significant correlations between the relative abundance of the ten major volatile compounds and antioxidant activity (*q* > 0.05), indicating that the observed antioxidant activity could not be attributed to any single major constituent based on the present data.

### 2.5. Antibacterial Activity

The antibacterial activities of 13 *Lamiaceae* EOs were evaluated against five opportunistic pathogens (Table 3). Inhibition zone diameters ranged from 6.00 to 38.13 mm, with NI (no inhibition) indicating no inhibition beyond the 6-mm paper disc. Overall, active oils showed stronger inhibition against Gram-positive than Gram-negative bacteria. Based on the inhibition zone results in the disc diffusion assay, four EOs showing relatively strong antibacterial activity, namely *Origanum vulgare*, *Thymus citriodorus*, *Thymus vulgaris*, and *Thymus mongolicus*, were selected from the 13 EOs for subsequent minimum inhibitory concentration (MIC) determination. Among these oils, *Thymus mongolicus* produced the largest inhibition zone against *S. haemolyticus* (38.13 ± 1.95 mm). The MIC results for the four selected oils (Table 4) showed values ranging from 2 to 8 µg/mL, with generally lower MICs observed for Gram-positive strains.

Against *E. coli*, *Thymus* oils showed strong activity, with *Thymus mongolicus* exhibiting the largest zone (23.43 mm), while *Mentha canadensis* and *Nepeta cataria* showed NI. MICs against *E. coli* were 2 µg/mL for *Origanum vulgare*, *Thymus citriodorus*, and *Thymus mongolicus*, and 4 µg/mL for *Thymus vulgaris*. Against *K. pneumoniae*, *Origanum vulgare* showed the lowest MIC (2 µg/mL), followed by *Thymus mongolicus* (4 µg/mL), while *Thymus citriodorus* and *Thymus vulgaris* showed higher MICs (8 µg/mL each). Against *S. marcescens*, *Origanum vulgare* showed the largest inhibition zone (15.17 mm), followed by *Thymus* oils (12.43–13.16 mm); MICs were 4 µg/mL for *Origanum vulgare* and *Thymus mongolicus*, and 8 µg/mL for *Thymus citriodorus* and *Thymus vulgaris*.

### 2.6. Cytotoxicity

The cytotoxic effects of 13 EOs derived from *Lamiaceae* species against human hepatocellular carcinoma-related HepG2 and prostate adenocarcinoma LNCaP cells were evaluated by determining their half-maximal inhibitory concentrations (IC_50_), with the results summarized in Table 5. Their cytotoxicity toward the corresponding normal cell lines, THLE-2 and RWPE-1, was further assessed (Table S3), and the selectivity index (SI) of each EO was calculated (Figure S3). The goodness-of-fit of the concentration–response curves was assessed using the coefficient of determination (*R*^2^), with the corresponding *R*^2^ values provided in Table S4.

All tested EOs exhibited IC_50_ values below 1.0 μg/mL against both cancer cell lines, although their in vitro selectivity varied among cell lines. Against HepG2 cells, *Thymus vulgaris* and *Elsholtzia ciliata* showed relatively low IC_50_ values of 0.35 and 0.36 μg/mL, respectively, whereas *Thymus mongolicus* showed the highest IC_50_ value (0.95 μg/mL) among the tested EOs (*p* < 0.05). The corresponding SI values for *T. vulgaris* and *E. ciliata* were 14.01 and 21.80, respectively, indicating greater in vitro selectivity toward HepG2 cells relative to the tested normal hepatocyte line. Against LNCaP cells, *E. ciliata*, *Scutellaria baicalensis*, and *Perilla frutescens* showed relatively low IC_50_ values of 0.31, 0.34, and 0.35 μg/mL, respectively. In contrast, *Origanum vulgare* and *Rosmarinus officinalis* showed relatively higher IC_50_ values of 0.83 and 0.81 μg/mL, respectively, with corresponding SI values of 12.58 and 10.03.

A comparison between the two cancer cell lines revealed cell line-dependent differences in EO sensitivity. *Origanum vulgare* and *Rosmarinus officinalis* showed approximately 1.6-fold higher IC_50_ values against LNCaP than against HepG2 cells, whereas *Schizonepeta tenuifolia* and *Thymus citriodorus* showed approximately 1.5-fold higher IC_50_ values against HepG2 than against LNCaP cells. Among the tested EOs, *Elsholtzia ciliata* and *Perilla frutescens* showed relatively low IC_50_ values across both cancer cell lines (0.31–0.40 μg/mL). Their corresponding SI values were 21.80 and 23.24 for HepG2, and 19.65 and 13.15 for LNCaP, respectively, indicating greater in vitro selectivity toward the tested cancer cell lines relative to the corresponding normal cell lines.

### 2.7. Correlation Analysis Between Volatile Compounds and Bioactivities

**Figure 3:**
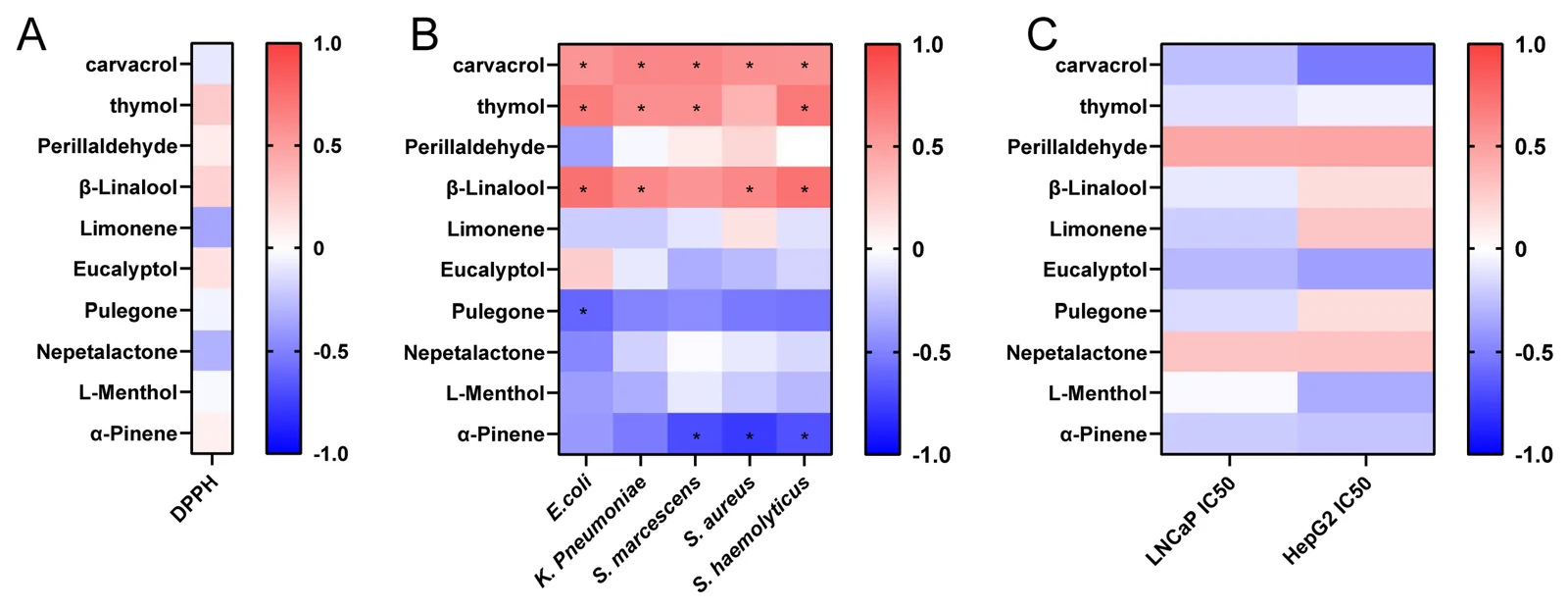
Spearman correlation heatmaps between the relative contents of ten major volatile compounds and bioactivity indices of 13 EOs. (**A**) DPPH radical scavenging activity (1/IC_50_). (**B**) Antibacterial activity against opportunistic bacteria (inhibition zone diameter, mm). (**C**) Cytotoxicity against LNCaP and HepG2 cells (1/IC_50_). The color scale represents Spearman’s correlation coefficient (ρ), with red indicating positive correlation and blue indicating negative correlation. *, FDR-adjusted *q* < 0.05 (Benjamini–Hochberg correction, n = 13 EO samples).

To further explore the potential chemical basis of the observed bioactivities, Spearman’s rank correlation analysis was performed between the relative contents of the ten major volatile compounds and the measured bioactivity indices (Figure 3). GC-MS-based phytochemical profiling combined with chemical composition–activity analysis has been used to explore potential relationships between plant metabolites and biological activities [22]. Because only 13 EOs were included, these analyses were considered exploratory and were used to identify associations rather than establish causal relationships. The complete Spearman correlation matrix, including correlation coefficients (ρ) and FDR-adjusted *q* values, is provided in Table S5.

For antioxidant activity, the strongest DPPH radical-scavenging activities were observed for *Perilla frutescens* and *Rosmarinus officinalis*, which were characterized by relatively high levels of perillaldehyde and eucalyptol, respectively. Other highly active oils, including *Thymus*, *Mentha*, and *Scutellaria*, were characterized by different dominant constituents. However, none of the ten major compounds showed a significant correlation with DPPH activity after Benjamini–Hochberg FDR correction (*q* > 0.05), indicating that the antioxidant activity could not be attributed to any single major constituent based on the present dataset.

In contrast, several volatile compounds were significantly associated with antibacterial activity [23]. Carvacrol was positively correlated with inhibition zones against *K. pneumoniae*, *S. marcescens*, and *S. aureus*, while thymol was positively correlated with *S. haemolyticus* and *E. coli*. β-Linalool was also positively correlated with *E. coli* and *S. aureus*. Conversely, α-pinene showed negative correlations with several bacterial strains. These findings identify carvacrol, thymol, and β-linalool as candidate constituents associated with antibacterial activity, although the correlations do not establish direct causality. For cytotoxicity, none of the ten major compounds showed significant correlations with IC_50_ values against HepG2 or LNCaP cells after FDR correction (*q* > 0.05).

Overall, the correlation analysis revealed activity-dependent associations between specific volatile constituents and antibacterial activity, whereas no significant associations were identified for antioxidant or cytotoxic activity. These findings suggest that the biological activities of the EOs cannot be consistently explained by the abundance of individual major constituents and may instead reflect differences in their overall chemical profiles.

### 2.8. EOs Alleviate Skin Inflammation by Reducing the Expression of Inflammation-Associated Molecules

**Figure 4:**
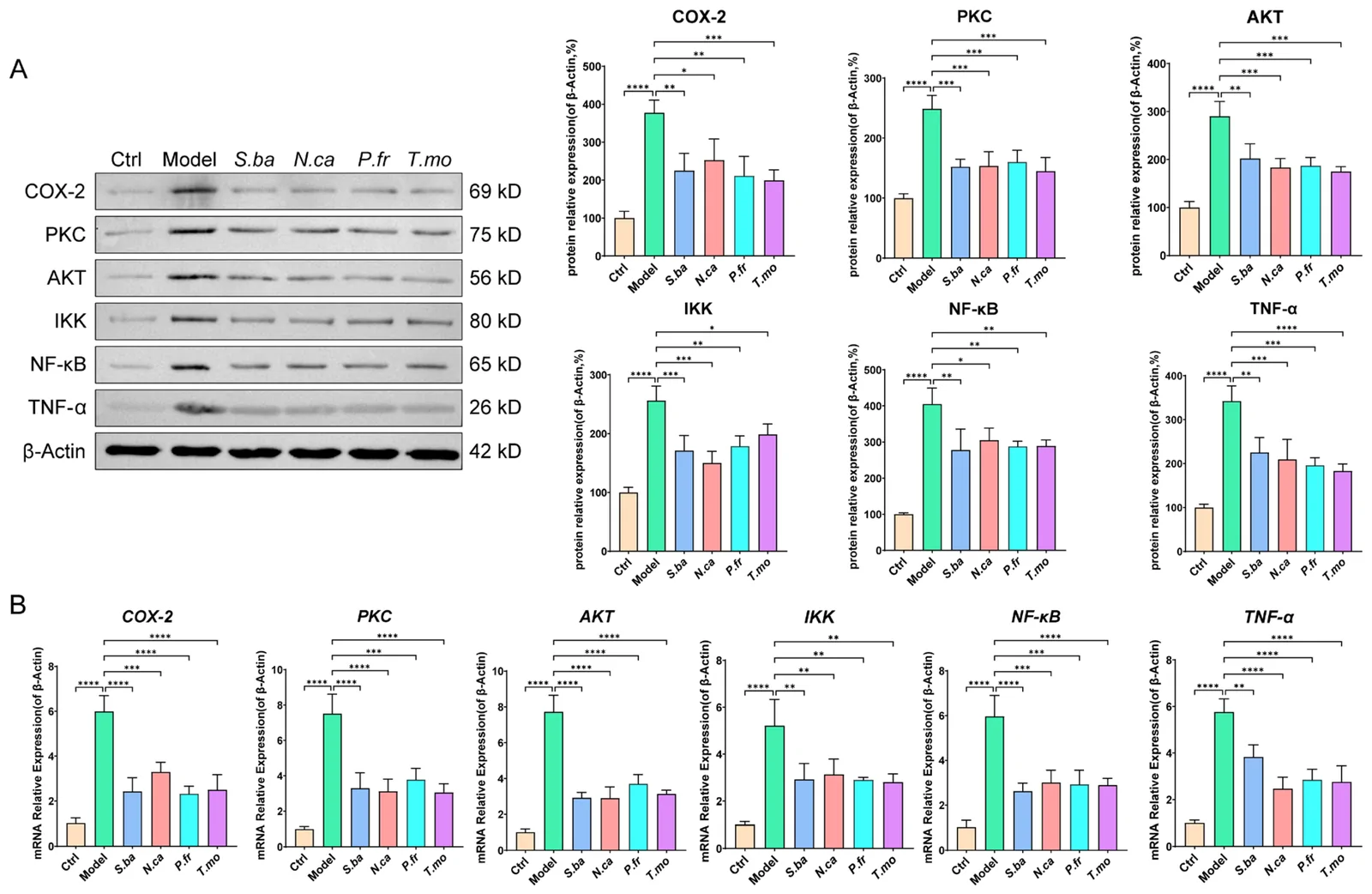
Inflammatory response in skin tissues after different treatments. (**A**) Western blot analysis of COX-2, TNF-α, NF-κB, IKK, Akt, and PKC protein expression, with corresponding semi-quantitative densitometric analysis. (**B**) qPCR analysis of the relative mRNA levels of *COX-2*, *TNF-α*, *NF-κB*, *IKK*, *Akt*, and *PKC*. Data are presented as mean ± SD (n = 6 mice per group). Different asterisks indicate significant differences among groups (* *p* < 0.05, ** *p* < 0.01, *** *p* < 0.001,**** *p* < 0.0001).

To evaluate the interventional effects of selected EOs on skin inflammation, we next investigated inflammatory signal transduction, the expression of inflammatory mediators, and histological damage. Western blotting revealed that when compared with the control group, the protein levels of Cyclooxygenase-2 (COX-2), tumor necrosis factor-alpha (TNF-α), nuclear factor-kappa B (NF-κB), IκB kinase (IKK), protein kinase B (Akt) and protein kinase C (PKC) proteins in skin tissues of the model group were significantly increased (*p* < 0.05) (Figure 4A and Figure S4A,B), thus suggesting that the combination of sodium dodecyl sulfate (SDS) and squalene successfully induced a significant local inflammatory response. The observed increase in COX-2 indicated the active synthesis of inflammatory-related lipid mediators while the increase in TNF-α indicated an enhanced local pro-inflammatory state. The synchronous upregulation of IKK, NF-κB, and Akt was associated with changes in inflammation-related signaling. Previous studies reported that COX-2 is an important inflammatory-related enzyme in skin inflammation, that TNF-α can amplify inflammatory responses through the NF-κB/MAPK pathway, and that IKK, as the core regulatory node of NF-κB activation, plays a key role in the transduction of inflammatory signals. Furthermore, the PI3K/Akt axis plays a key role in the maintenance of skin homeostasis along with the occurrence and development of various non-tumorous skin diseases [24,25].

Compared with the model group, the expression levels of COX-2, PKC, Akt, IKK, NF-κB, and TNF-α proteins in each EO intervention group decreased to varying degrees (*p* < 0.05) (Figure 4A and Figure S4C,D). Of these, the reduction in COX-2 and TNF-α indicated that EO intervention could reduce the generation of inflammatory mediators and the intensity of pro-inflammatory responses. The downregulation of PKC, Akt, IKK, and NF-κB was associated with reduced expression of inflammation-related signaling molecules. Collectively, these results indicated that different EOs were associated with reduced expression of inflammatory mediators and inflammation-related signaling molecules. Previous studies also showed that some EOs downregulated COX-2, TNF-α and NF-κB in a model of skin inflammation, accompanied by an improvement in the inflammatory phenotype, which is consistent with the changes observed in this study [26].

Results arising from Quantitative Polymerase Chain Reaction (qPCR) confirmed the trends observed in our Western blotting analysis. Compared with the control group, the mRNA levels of *COX-2*, *PKC*, *Akt*, *IKK*, *NF-κB* and *TNF-α* in the model group were significantly increased (*p* < 0.05, Figure 4B and Figure S5), indicating that the inflammatory activation induced by the model occurred not only at the protein level but also at the transcriptional level. Following intervention with different EOs, the mRNA levels of each inflammatory-related gene were lower than those in the model group (*p* < 0.05), suggesting that EOs were associated with reduced transcription of inflammation-related genes. The changes in protein and mRNA levels were broadly consistent, supporting the association of EO treatment with reduced expression of inflammation-related molecules. Previous studies on the anti-inflammatory effects of EOs also suggested that the effects of EOs are often accompanied by a reduction in *NF-κB* activation and a reduction in the expression of downstream pro-inflammatory cytokines.

**Figure 5:**
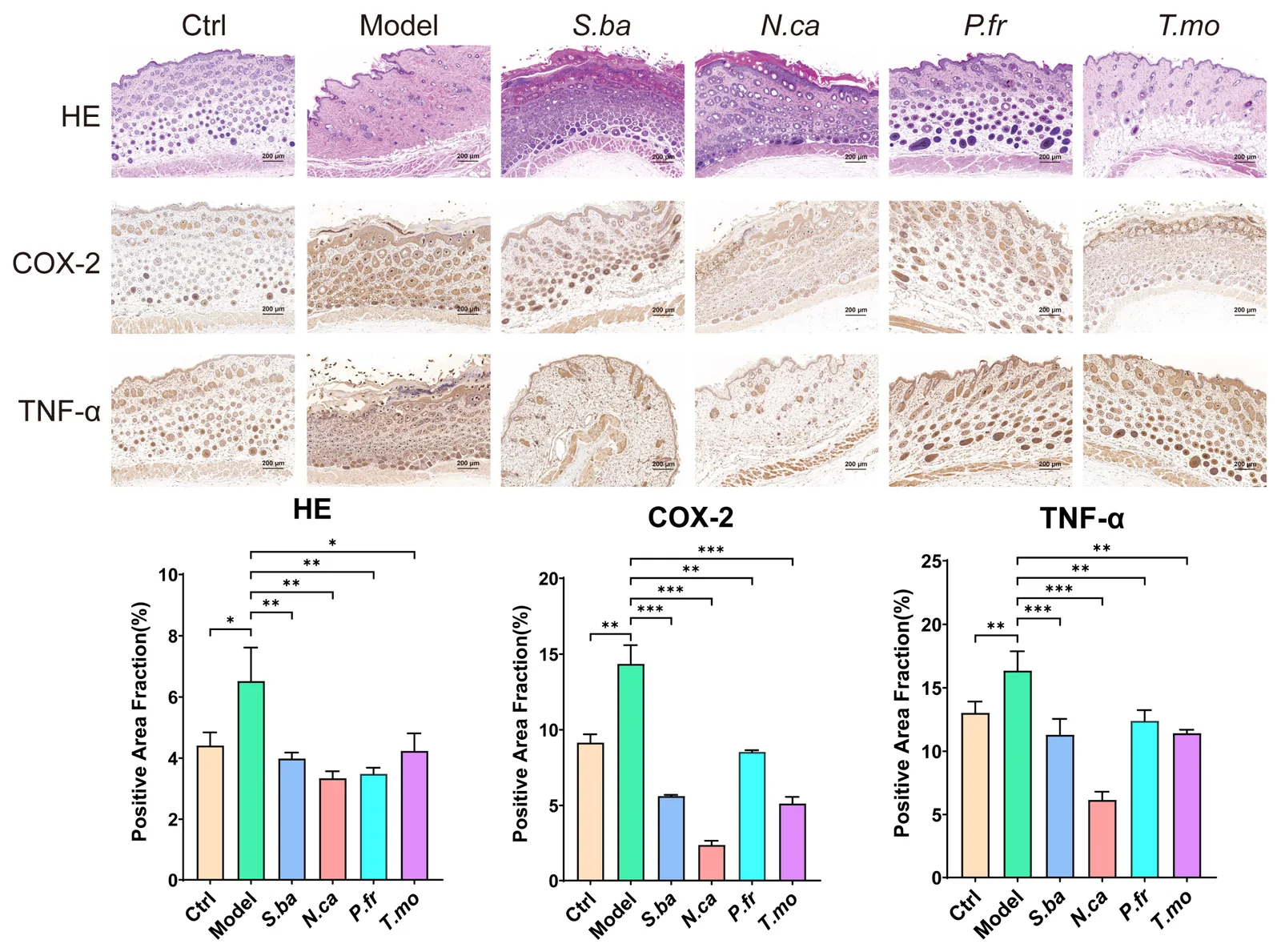
Representative H&E-stained sections for histopathological evaluation and immunohistochemical staining of COX-2 and TNF-α, with corresponding semi-quantitative analysis. Data are presented as mean ± SD. Data are presented as mean ± SD (n = 6 mice per group). Different letters or asterisks indicate significant differences among groups (* *p* < 0.05, ** *p* < 0.01, *** *p* < 0.001).

To further validate the anti-inflammatory effects at the tissue level, we next examined histopathological changes and localized expression of inflammatory mediators via histological and immunohistochemical analyses (Figure 5 and Figure S6).

Histological analysis further revealed that the structure of the skin tissue in the control group was intact, with uniform epidermal thickness and clear layers with no obvious abnormalities. However, in the model group, the epidermis was significantly thickened (*p* < 0.05), with abnormal keratinization and increased inflammatory cell infiltration (Figure 5). Thickening of the epidermis and abnormal keratinization reflected an imbalance in epidermal proliferation and differentiation under inflammatory conditions, while the increase in inflammatory cell infiltration indicated enhanced recruitment of local inflammatory cells, suggesting that the model group had suffered from obvious inflammatory tissue damage. Following intervention with different EOs, the epidermal thickening was reduced, the tissue structure became more regular, and inflammatory cell infiltration was decreased (*p* < 0.05), indicating that EO treatment was associated with attenuation of inflammation-related histopathological damage. Previous studies have reported that in models of skin inflammation or dermatitis, increased expression of inflammatory molecules such as TNF-α and COX-2 is often accompanied by epidermal thickening and infiltration of inflammatory cells, and that reduced expression of these inflammatory molecules is associated with concomitant improvement in histological damage [24].

Immunohistochemistry analysis further validated the changes in the levels of key inflammatory molecules at the level of tissue localization. Compared with the control group, the positive staining of COX-2 and TNF-α in skin tissues from the model group was significantly enhanced (*p* < 0.05), suggesting that the expression of inflammatory mediators in local lesions was significantly increased. Following intervention with different EOs, the expression levels of COX-2 and TNF-α were both weaker than those in the model group (*p* < 0.05), indicating that EO intervention reduced the local expression levels of inflammation-related molecules in lesions. These results were consistent with those derived from Western blotting and qPCR analyses.

In conclusion, the analysis shows that the use of SDS in mice caused significant local skin inflammation, manifested by the activation of inflammatory signaling molecules, increased expression of inflammatory mediators, and obvious pathological tissue damage. After being intervened with different EOs, these abnormal changes were reversed to varying degrees. These findings suggest that treatment with different EOs was associated with attenuation of inflammatory and histopathological changes, accompanied by reduced expression of inflammation-related molecules and inflammatory mediators.

## 3. Discussion

GC-MS analysis demonstrated substantial chemical heterogeneity among the 13 *Lamiaceae* EOs. The identified constituents mainly comprised monoterpene hydrocarbons, oxygenated monoterpenes, sesquiterpene hydrocarbons, oxygenated sesquiterpenes, and phenolic monoterpenes, although their relative contributions varied considerably among species. This finding is consistent with previous comparative investigations showing marked differences in the chemical composition of EOs among multiple *Lamiaceae* species [15,16]. Such chemical diversity provides an important basis for interpreting the distinct biological activity profiles observed among the EOs examined in the present study.

At the level of individual constituents, several species displayed characteristic dominant compounds. *Origanum vulgare* showed a carvacrol-rich profile, consistent with previous reports describing the chemical composition of *O. vulgare* EO [11]. The EO of *Rosmarinus officinalis* was characterized mainly by eucalyptol and α-pinene, broadly consistent with previously reported chemical profiles of rosemary EO [12]. The *Thymus* species examined in the present study showed thymol-rich profiles, which is also in agreement with the occurrence of phenolic monoterpenes as characteristic constituents reported for other *Thymus* EOs [27]. Other species exhibited distinct dominant constituents, including limonene in *Scutellaria baicalensis*, α-muurolene in *Scutellaria scordifolia*, L-menthol in *Mentha canadensis*, perillaldehyde in *Perilla frutescens*, nepetalactone in *Nepeta cataria* and *Elsholtzia ciliata*, pulegone in *Phlomis mongolica*, and anethole- and anisaldehyde-related constituents in *Schizonepeta tenuifolia*. Collectively, these species-specific chemical profiles highlight the considerable chemical diversity of the Lamiaceae EOs and provide a phytochemical framework for interpreting their subsequent differences in antioxidant, antibacterial, cytotoxic, and anti-inflammatory activities.

In the DPPH assay, *Rosmarinus officinalis* exhibited the highest free radical scavenging activity, followed by *Perilla frutescens* and *Scutellaria baicalensis*. IC_50_ determination further confirmed that *Perilla frutescens* and *Rosmarinus officinalis* possessed the strongest antioxidant potency, followed by *Thymus mongolicus*, *Mentha canadensis*, and *Scutellaria baicalensis*. Although these EOs were characterized by different major constituents, including oxygenated monoterpenes (eucalyptol and perillaldehyde) and phenolic monoterpenes (thymol and carvacrol), Spearman correlation analysis after FDR correction revealed no significant individual compound–activity correlations (*q* > 0.05). Therefore, the present data do not support attributing antioxidant activity to any single major compound. The observed differences may instead reflect the overall chemical composition of each EO. The relatively large SDs observed for some EOs in the DPPH assay may reflect the intrinsic compositional complexity and volatility of EO samples, as well as potential differences in dispersion within the reaction system.

Regarding antibacterial activity, *Thymus mongolicus* and *Origanum vulgare*, characterized by relatively high proportions of thymol and carvacrol, respectively, exhibited the strongest inhibitory effects against both Gram-positive (*S. aureus*) and Gram-negative (*E. coli*, *K. pneumoniae*, and *S. marcescens*) bacteria, with MIC values ranging from 2 to 8 µg/mL. The antibacterial properties of phenolic monoterpenes have been associated with their ability to interact with and disrupt bacterial membranes [28]. Furthermore, the differential susceptibility of Gram-positive and Gram-negative bacteria may also reflect differences in bacterial envelope structure [29]. Consistent with these observations, carvacrol and thymol showed significant positive associations with inhibition zones against several bacterial strains in the present dataset. However, these correlations were exploratory and do not establish a direct causal contribution of individual compounds to antibacterial activity.

Regarding cytotoxicity, all tested EOs exhibited potent activity against HepG2 and LNCaP cancer cell lines. *Elsholtzia ciliata* and *Perilla frutescens* showed relatively strong activity across both tested cancer cell lines, together with differential selectivity toward cancer and normal cells. In contrast, *Origanum vulgare* and *Rosmarinus officinalis*, despite their excellent antioxidant and antibacterial activities, exhibited relatively weaker cytotoxicity. These differences in activity patterns further highlight the activity-dependent biological profiles of the tested EOs.

An exploratory positive association was observed between antioxidant capacity and anti-inflammatory activity. EOs with higher DPPH scavenging rates, such as those from *Rosmarinus officinalis* and *Perilla frutescens*, were more strongly associated with reduced expression of NF-κB, TNF-α, and COX-2. Consistent with previous reports that phenolic-rich *Perilla* extracts exert anti-inflammatory effects via downregulating TNF-α-induced NF-κB activation and COX-2 expression [30], our findings showed an association between antioxidant capacity and reduced expression of inflammation-related molecules, although a direct mechanistic relationship could not be established. In the SDS-induced murine skin inflammation model, the model group exhibited significantly elevated levels of COX-2, TNF-α, NF-κB, IKK, Akt, and PKC, along with epidermal thickening and inflammatory infiltration, confirming successful model establishment. EO intervention downregulated these molecules, restored epidermal thickness, and alleviated inflammation, as consistently confirmed by Western blotting, qPCR, and immunohistochemistry. The concurrent changes in NF-κB, IKK, COX-2, and TNF-α expression were associated with attenuation of inflammation-related responses. Collectively, these findings suggest that Lamiaceae EOs were associated with reduced expression of multiple inflammation-related molecules and attenuation of SDS-induced skin inflammatory responses.

Taken together, the 13 *Lamiaceae* EOs showed distinct chemical profiles accompanied by diverse patterns of biological activity. The standardized comparative evaluation across multiple genera allowed these chemical and biological differences to be considered within a common experimental framework. *Origanum vulgare* and *Thymus mongolicus* showed relatively strong antibacterial activity, particularly in terms of their low MIC values, whereas *Rosmarinus officinalis* and *Perilla frutescens* showed strong antioxidant activity. *Elsholtzia ciliata* and *Perilla frutescens* exhibited relatively strong cytotoxic activity across the tested cancer cell lines. These findings provide a comparative basis for further investigation of the chemical constituents associated with specific bioactivities of *Lamiaceae* EOs.

Nevertheless, several limitations should be acknowledged. First, the SDS-induced murine skin inflammation model lacked a positive control, such as hydrocortisone, which limited direct comparison with an established anti-inflammatory treatment. In addition, dose–response and pathway-specific intervention studies were not performed, and therefore the present findings do not establish the direct mechanisms underlying the observed anti-inflammatory effects. Second, although a standardized hydrodistillation procedure was used, potential losses of volatile constituents during plant material handling and extraction cannot be completely excluded, particularly because glandular trichomes on *Lamiaceae* leaves may release volatile components during processing. Thus, the chemical profiles reported here represent the composition obtained under the present extraction conditions and may not fully reflect the native or commercial EO composition. Third, commercially available EOs were not included as reference materials, limiting direct comparison between the experimental samples and marketed products. Finally, the correlation analyses were exploratory and cannot establish causal relationships between individual constituents and bioactivities, while the contribution of individual compounds within complex EO mixtures could not be distinguished. Future studies incorporating appropriate positive controls, commercial reference oils, standardized quality-control procedures, and purified or defined constituents, together with dose–response and pathway-specific validation, are warranted to further clarify the reproducibility and biological relevance of these findings.

## 4. Materials and Methods

### 4.1. Plant Materials

Thirteen *Lamiaceae* species at the full flowering stage, with consistent growth conditions and the same number of growth years, were selected from the Experimental Base of Inner Mongolia Agricultural University in August 2025. The plant materials were taxonomically identified by Professor Li Hong from Inner Mongolia Agricultural University based on their morphological characteristics and relevant taxonomic references.

### 4.2. Extraction of EO

Fresh above-ground parts of the plants were washed, dried in the shade, then ground and sieved immediately before hydrodistillation to minimize the loss of volatile components. Then, 50 g of the dried and uniformly powdered plant material was accurately weighed and placed in a round-bottomed flask, and 800 mL of distilled water and 3–5 zeolite boiling stones were added. The flask was then heated until the water boiled and generated steam, which contained vaporized EOs. The extraction temperature was maintained at approximately 100 °C. The mixture was maintained at a gentle boil for 3–4 h, and the steam was condensed through a water-cooled condenser and collected in a receiving flask to form a milky oil–water emulsion. After completion of the distillation, the collected emulsion was transferred to a separatory funnel and allowed to stand until the oil and aqueous phases separated. The aqueous phase was removed through the stopcock of the separatory funnel, and the oil phase was collected separately. The collected oil layer was dehydrated by the addition of approximately 1–2 g of anhydrous sodium sulfate and allowed to stand until the oil phase became clear. The dried oil was then filtered, transferred to amber glass vials, sealed, and stored at 4 °C in the dark until further analysis. The EO yield (%) was determined as the ratio of the extracted oil mass to the dry weight of the aerial parts of the plant, multiplied by 100. Three independent extractions were performed for each sample, and the results are expressed as mean ± standard deviation (SD) [31].

Conventional hydrodistillation was applied to all 13 *Lamiaceae* species using the same sample mass, water-to-sample ratio, extraction time (3–4 h), and downstream processing procedure to ensure methodological consistency and facilitate comparison of EO yields and chemical profiles among species [32].

### 4.3. EO Analysis

EOs were analyzed using a DSQ-II ultra (Thermo Electron, USA) GC-MS instrument (Agilent, Santa Clara, CA, USA) with a DB-5MS capillary column (0.25 mm × 30 m, 0.25 μm film thickness). The operating conditions were as follows: the GC temperature program commenced at 40 °C for 1 min, increased at 5 °C/min from 40 °C to 280 °C, and then remained at 280 °C for 5 min. The carrier gas was helium with a flow rate of 1.0 mL/min; the injection temperature was 250 °C; the injection volume was 1 μL; and the split ratio was 100:1. The mass spectrometry conditions were as follows: the pre-column pressure was 70 kPa; the electron energy was 70 eV; and the ion source temperature was 230 °C. The RI of each compound was calculated using the van den Dool and Kratz equation for linearly temperature-programmed gas chromatography, based on the n-alkane standard substances (C_6_–C_40_) [33]. The RI was calculated according to the following equation:

RI = 100[n + (t_R_(x) − t_R_(n))/(t_R_(n + 1) − t_R_(n))]

where t_R_(x) is the retention time of the compound of interest, t_R_(n) and t_R_(n + 1) are the retention times of the n-alkanes eluting immediately before and after the compound, respectively, and n is the carbon number of the preceding n-alkane.

Compound identification was achieved by comparing the mass spectra with the National Institute of Standards and Technology mass spectral library, in conjunction with RI matching. Compounds were tentatively identified when the mass spectrum match factor was ≥80% and the experimental RI was consistent with the literature RI. Additionally, squalene was analyzed as an external reference compound to confirm the identification of triterpene-related compounds and to verify the stability of the chromatographic system; it was not used for compound-specific quantitative calibration.

The chemical composition of the EOs was evaluated using relative peak-area normalization. The relative content of each identified compound was calculated as the peak area of the individual compound divided by the total peak area of all identified compounds and expressed as a percentage:

Relative content (%) = (peak area of an individual compound/total peak area of all identified compounds) × 100.

These values represent the relative abundance of individual constituents within each EO rather than absolute concentrations.

### 4.4. HCA and PCA

Based on the GC-MS chemical profiles of the 13 EOs, HCA and PCA were performed to visualize the chemical variation among the samples. The data matrix consisted of 13 samples (rows) × 51 identified compounds (columns), with the relative content (%) of each compound used as variables. A single representative EO sample was analyzed for each species, and the relative content of each compound was averaged from triplicate injections. For compounds not detected (below the limit of detection) in some samples, a value of 0.0001% was assigned to ensure data completeness. Prior to multivariate analysis, all content data were Z-score standardized to ensure equal contribution of all variables, regardless of their absolute abundances; no logarithmic transformation was applied. HCA was performed using Euclidean distance as the similarity measure and Ward’s linkage method for dendrogram construction. The dendrogram was generated using OriginPro 2024 (OriginLab Corporation, Northampton, MA, USA), and the heatmap was generated using GraphPad Prism software 10.1.2 (GraphPad Software, San Diego, CA, USA). PCA was also conducted using Origin software, with the first four principal components (PC1–PC4; eigenvalues > 1) extracted. Variable loadings were calculated to assess the contribution of individual compounds to the principal components, and the cumulative variance explained was reported. Given the limited sample size (n = 13) relative to the number of variables (*p* = 51), the multivariate analyses were interpreted as exploratory. Together, HCA and PCA provide complementary visualizations of chemical variation within the same dataset and facilitate exploratory characterization of chemotype patterns.

### 4.5. Antioxidant Activity Assay

The antioxidant activity of 13 EOs from the *Lamiaceae* family was determined using the DPPH free radical scavenging capacity assay. Each EO sample was freshly prepared in anhydrous ethanol before the experiment. A single concentration screening experiment was conducted, where the EO samples were diluted to a working concentration of 2 μg/mL. 400 μL of the EO solution was mixed with 600 μL of the DPPH working solution and placed in a measurement tube for 30 min at 25 °C in the dark. Then, the mixture was centrifuged at 4000 rpm for 5 min, and 800 μL of the supernatant was measured at a wavelength of 517 nm to determine the absorbance. The absorbance was recorded after zeroing with anhydrous ethanol. Trolox and methanol were used as positive and negative controls, respectively. Three independent experiments were performed, and the results are presented as mean ± SD. After confirming that the EOs had antioxidant activity at a concentration of 2 μg/mL, a series of concentration gradients were set for the samples to determine the IC_50_ value. DPPH free radical scavenging rate was determined using Equation:

DPPH free radical scavenging rate (%) = [1 − (A_sample_ − A_sample blank_)/Control] × 100

In Equation, Control represents the absorbance of the DPPH solution without the oil sample; A_sample_ represents the absorbance of the DPPH solution containing the test oil sample; and A_sample blank_ represents the absorbance of the oil sample without the DPPH solution [34,35,36].

### 4.6. Antibacterial Activity Assay

The antibacterial activity of EOs from 13 *Lamiaceae* species was evaluated using the filter paper diffusion method, and the inhibition zone diameters were measured in millimeters. The test strains were *Staphylococcus aureus* (ATCC 6538), *Escherichia coli* (ATCC 25922), *Serratia marcescens* (ATCC 14041), *Staphylococcus haemolyticus* (ATCC 29970), and *Klebsiella pneumoniae* (ATCC 13883). Bacterial cultures were adjusted to a concentration of 1 × 10^6^ CFU/mL, and 150 μL aliquots were evenly spread onto LB agar plates. Sterile filter paper discs (diameter: 6 mm) impregnated with 2 μL of each EO sample were then placed on the surface of the inoculated agar plates. Following incubation at 37 °C for 24 h, the diameters of the inhibition zones were measured using a vernier caliper for each EO, and three independent experiments were performed for each EO–bacterial strain combination [27,37]. A disc diameter of 6 mm indicated no noticeable inhibitory activity, whereas any inhibition zone diameter greater than 6 mm was considered to indicate antimicrobial effectiveness.

Based on the results of the paper disc diffusion assay, four EOs (*Origanum vulgare*, *Thymus citriodorus*, *Thymus vulgaris*, and *Thymus mongolicus*) exhibiting the largest inhibition zone diameters among the 13 tested EOs were selected for subsequent minimum inhibitory concentration (MIC) determination. The final bacterial inoculum in each well of the 96-well microplate was approximately 5 × 10^5^ CFU/mL. The EOs were subjected to two-fold serial dilution to obtain final concentrations of 32, 16, 8, 4, 2, 1, and 0.5 μg/mL in the wells. Gentamicin was used as the positive control and was tested at final concentrations of 8, 4, 2, 1, 0.5, 0.25, and 0.125 μg/mL, 10% ethanol containing 1% Tween-80 was used as the solvent negative control, cation-adjusted Mueller–Hinton broth (CAMHB) without EO was used as the growth control, and uninoculated CAMHB was used as the blank control. After incubation at 37 °C for 16–18 h, the MIC was defined as the lowest EO concentration at which no visible bacterial growth was observed at the bottom of the well.

### 4.7. Cytotoxicity Assay

The cytotoxicity of the 13 EOs was evaluated using the Cell Counting Kit-8 (CCK-8) assay, with LNCaP, HepG2, THLE-2, and RWPE-1 as test models. The cell lines used were all purchased from Zhejiang Meisen Cell Technology Co., Ltd. (Hangzhou, China). HepG2, LNCaP, THLE-2, and RWPE-1 cells were seeded into 96-well plates at a density of 1 × 10^4^ cells per well. The plates were then incubated at 37 °C in 5% CO_2_ to allow cell attachment. Following adhesion, the experimental groups were treated with the test EOs diluted in the culture medium at a starting concentration of 1 μg/mL, followed by two-fold serial dilutions to obtain concentrations of 0.5, 0.25, 0.125, and 0.0625 μg/mL, while the control group cells were only treated with the culture medium. After 72 h of incubation, 10 μL of CCK-8 solution was added to each well, and the cells were incubated for another 2 h. Subsequently, absorbance was measured at 450 nm using a microplate reader. Cell inhibition rate (%) was calculated using Equation.

Cell inhibition rate (%) = [(Ac − As)/(Ac − Ab)] × 100

In Equation, As = absorbance of the test well (the absorbance of the well containing cells, culture medium, CCK-8, and the test compound); Ab = absorbance of the blank well (the absorbance of the well containing culture medium and CCK-8); and Ac = absorbance of the control well (the absorbance of the well containing cells, culture medium, and CCK-8) [38,39].

The IC_50_ values were calculated by nonlinear regression using a four-parameter logistic model with a variable slope in GraphPad Prism and expressed as mean ± SD from three independent experiments. The selectivity index (SI) was calculated as follows:

SI = IC50 (normal cell line)/IC50 (corresponding cancer cell line).

THLE-2 and RWPE-1 were used as the corresponding normal cell lines for HepG2 and LNCaP, respectively.

### 4.8. Spearman’s Rank Correlation Analysis

To evaluate the relationships between the major volatile compounds and the bioactivity parameters, Spearman’s rank correlation analysis was performed on the 13 EO samples using GraphPad Prism software. The bioactivity indices included DPPH scavenging activity (1/IC_50_), antibacterial activity (inhibition zone diameter, mm), and cytotoxicity (1/IC_50_ against HepG2 and LNCaP cells). A positive correlation coefficient indicates that higher compound content is associated with enhanced activity, whereas a negative coefficient indicates the opposite. *p*-values were adjusted for multiple testing using the Benjamini–Hochberg FDR method, and FDR-adjusted *p* < 0.05 was considered statistically significant (n = 13).

### 4.9. Establishment of a Murine Skin Inflammation Model and EO Intervention

The anti-inflammatory activities of the 13 EOs were evaluated using a mouse model of dorsal skin inflammation induced by topical application of SDS. All animal experimental procedures were reviewed and approved by the Animal Experiment Center of Inner Mongolia Agricultural University (Approval No. NND2025065). Eight-week-old female Balb/c mice weighing 20.0 ± 1.6 g were used in this study. Prior to the experiment, all mice were acclimatized for one week under specific pathogen-free conditions with free access to food and water, and constant environmental temperature, humidity, and light cycle were maintained to ensure physiological stability. After one week of acclimatization, the dorsal hair of the mice was shaved, and 100 μL of 10% SDS solution was topically applied once daily for three consecutive days to induce inflammation. After successful induction, SDS application was stopped, and the mice were randomly divided into the control group, the model group, and the 13 kinds of EO treatment groups (1%, *v*/*v*), with random number tables for grouping, with six mice in each group. Each EO was dissolved in a solubilizing system consisting of 10% ethanol and 1% Tween-80, and the solution was diluted with sterile physiological saline to a final volume of 10 mL to prepare a 1% (*v*/*v*) working solution. In the intervention groups, 100 μL of the corresponding EO preparation was topically applied to the inflamed area twice daily for 7 consecutive days, while the model group received an equal volume of the vehicle solution. The administration time, site, and dosage were consistent across all groups. After the final administration, the mice were deeply anesthetized by intraperitoneal injection of an overdose of sodium pentobarbital and euthanized by cervical dislocation. Dorsal skin tissues from the inflamed areas were subsequently collected. A portion of the tissue samples was fixed in 4% paraformaldehyde for histological examination, while the remaining tissues were immediately frozen in liquid nitrogen and stored at −80 °C for subsequent molecular analyses [40,41].

### 4.10. Quantitative Real-Time PCR

Total RNA was extracted from mouse skin samples using TRIzol Reagent (Invitrogen, Carlsbad, CA, USA). The concentration and purity of the extracted RNA were determined using a NanoDrop 2000 spectrophotometer (Thermo Fisher Scientific, Wilmington, DE, USA). Subsequently, complementary DNA (cDNA) was synthesized using the GoScript Reverse Transcription System (Promega, Madison, WI, USA). Quantitative Real-Time Polymerase Chain Reaction (qRT-PCR) was performed on an ABI QuantStudio 5 Real-Time PCR System (Applied Biosystems, Foster City, CA, USA) using GoTaq qPCR Master Mix (Promega, Madison, WI, USA). The 20 μL reaction mixture consisted of 2 × Master Mix (10 μL), forward and reverse primers (0.4 μL each), cDNA template (2 μL), and nuclease-free water (7.2 μL). The amplification protocol was as follows: initial denaturation at 95 °C for 30 s, followed by 40 cycles of denaturation at 95 °C for 5 s and annealing/extension at 60 °C for 30 s. After amplification, melt curve analysis was performed to verify the specificity of the amplified products. β-actin was used as the internal reference gene, and the relative expression levels of target genes were calculated using the 2^−ΔΔCt^ method [42]. The primer sequences for all genes are listed in Table S6 [43].

### 4.11. Western Blot Analysis

Dorsal skin tissues were collected from mice in each group and homogenized in precooled radioimmunoprecipitation assay (RIPA) lysis buffer supplemented with protease inhibitors on an ice-bath. Homogenates were then centrifuged at 12,000× *g* for 15 min at 4 °C, and the supernatants were collected. Total protein concentration was determined using a bicinchoninic acid protein assay kit (Beyotime Biotechnology, Shanghai, China), with β-actin as the internal reference protein. Semi-quantitative analysis of band intensity was performed using ImageJ (version 1.54k, Wayne Rasband, National Institutes of Health, Bethesda, MD, USA; https://imagej.net), and the relative expression levels of each target protein were expressed as the ratio of the grayscale intensity of the target protein to that of the internal reference protein [44]. The primary antibodies used were as follows: anti-COX-2 (1:1000, ab179800, Abcam, UK), anti-PKC (1:1000, ab32376, Abcam, UK), anti-IKK (1 μg/mL, ab7891, Abcam, UK), anti-TNF-α (1:1000, ab183218, Abcam, UK), anti-NF-κB (1:1000, ab32536, Abcam, UK), anti-AKT (1:2000, R380617, ZEN-BIO, China), and anti-β-actin (1:1000, ab8227, Abcam, UK). The secondary antibodies used were goat anti-mouse IgG H&L (HRP) (1:2000, ab97023, Abcam, UK) and goat anti-rabbit IgG H&L (HRP) (1:2000, ab205718, Abcam, UK).

### 4.12. Histopathological Analysis

Dorsal skin tissues were collected from mice in each group and fixed in 4% paraformaldehyde (Solarbio, Beijing, China) for 24 h. After routine dehydration, clearing, and paraffin embedding, the tissues were sectioned at a thickness of 4 μm. For hematoxylin and eosin (H&E) staining, the sections were deparaffinized in xylene, rehydrated through a graded series of ethanol concentrations, and then stained with hematoxylin and eosin. Following dehydration, clearing, and mounting, the sections were observed and imaged using a Nikon microscope system (Nikon, Tokyo, Japan). Histopathological changes, including epidermal thickness, the degree of keratinization, acanthosis, dermal edema, and inflammatory cell infiltration, were also evaluated to assess the effects of different treatments on inflammatory skin injury, as described previously [45].

For immunohistochemical, paraffin sections were deparaffinized and rehydrated, followed by antigen retrieval in citrate buffer (pH 6.0). After natural cooling, the sections were washed three times with PBS for 5 min each. Endogenous peroxidase activity was blocked with 3% hydrogen peroxide for 10 min at room temperature, followed by blocking with 5% BSA for 30 min at room temperature. After removal of the blocking solution, the sections were incubated with the appropriate primary antibodies overnight at 4 °C. On the following day, after PBS washing, the sections were incubated with the corresponding secondary antibodies for 30 min at room temperature. The immunoreactivity was then visualized using DAB, followed by hematoxylin counterstaining. After dehydration, clearing, and mounting, the sections were observed and imaged using a Nikon microscope system (Nikon, Japan) to evaluate the positive expression of target proteins. According to the experimental design, inflammation-related proteins in skin tissues were detected, and semi-quantitative analysis of the positive staining area or mean optical density was performed using ImageJ software.

### 4.13. Statistical Analysis

All experiments were performed with at least three independent biological replicates, and results are presented as mean ± SD. Technical replicates are specified in the corresponding method sections. Prior to statistical analysis, normality and homogeneity of variances were assessed. Data satisfying both assumptions were analyzed by one-way analysis of variance (ANOVA), and SPSS version 13.0 software (SPSS Inc., Chicago, IL, USA) was employed for Duncan’s multiple range test (*p* < 0.05) to determine the significant differences between groups. Data violating either assumption were analyzed using non-parametric tests.

## 5. Conclusions

This study provided a comparative evaluation of the chemical composition and multiple biological activities of essential oils from 13 *Lamiaceae* species under standardized experimental conditions. GC-MS analysis revealed substantial interspecific variation in volatile composition. *Origanum vulgare* was characterized by carvacrol and limonene; *Thymus* spp. by thymol; *Rosmarinus officinalis* by eucalyptol and α-pinene; *Mentha canadensis* by L-menthol; *Perilla frutescens* by perillaldehyde; and *Nepeta cataria* and *Elsholtzia ciliata* by nepetalactone. *Scutellaria baicalensis*, *Phlomis mongolica*, and *Schizonepeta tenuifolia* were characterized by limonene and isopulegone, pulegone, and anethole and anisaldehyde, respectively. Multivariate analysis separated the 13 oils into three chemotype groups: a carvacrol-rich *Origanum* group, a *Scutellaria* and *Thymus* group enriched in sesquiterpenes or thymol, and a group of the remaining six genera dominated by shared monoterpene hydrocarbons. The EOs also showed diverse antioxidant, antibacterial, cytotoxic, and anti-inflammatory activities. *Perilla frutescens* and *Rosmarinus officinalis* exhibited strong antioxidant activity, while *Thymus mongolicus* and *Origanum vulgare* showed notable antibacterial activity. *Elsholtzia ciliata* and *Perilla frutescens* displayed relatively strong cytotoxic activity against the tested cancer cell lines, and selected EOs attenuated SDS-induced inflammatory responses in mice. Correlation analyses did not support attributing antioxidant activity to any single major compound, highlighting the potential contribution of the overall chemical composition of EO mixtures. These findings provide a comparative basis for further investigation of *Lamiaceae* EOs and their bioactive constituents. Future studies using purified compounds, standardized EO preparations, appropriate positive controls, and mechanistic validation are warranted to clarify the contributions of individual constituents and their interactions to the observed biological activities.

## Figures and Tables

**Table 1:** Source of materials, extraction site, color and yield of EOs for 13 species of the *Lamiaceae* family in China.

Latin Name	Source Area of Experimental Materials	The Extraction Site of EOs	Color of EO	Oil Yield (wt %)
*Rosmarinus officinalis*	Harbin	Branches and leaves	Faint yellow	1.79 ± 0.65
*Scutellaria baicalensis*	Inner Mongolia	Branches and leaves	Yellow	1.83 ± 0.68
*Nepeta cataria*	Shandong	Branches and leaves	Faint yellow	3.12 ± 0.92
*Mentha canadensis*	Shanxi	Branches and leaves	Yellow	2.13 ± 0.63
*Perilla frutescens*	Harbin	Branches and leaves	Faint yellow	2.16 ± 0.54
*Elsholtzia ciliata*	Harbin	Branches and leaves	Faint yellow	2.84 ± 0.48
*Origanum vulgare*	Shandong	Branches and leaves	Reddish yellow	3.16 ± 0.71
*Scutellaria scordifolia*	Shanxi	Branches and leaves	Yellow	1.52 ± 0.82
*Phlomis mongolica*	Inner Mongolia	Branches and leaves	Faint yellow	1.47 ± 0.76
*Schizonepeta tenuifolia*	Inner Mongolia	Branches and leaves	Faint yellow	1.73 ± 0.88
*Thymus citriodorus*	Shandong	Branches and leaves	Faint yellow	1.50 ± 0.82
*Thymus vulgaris*	Shandong	Branches and leaves	Yellow	1.50 ± 0.79
*Thymus mongolicus*	Inner Mongolia	Branches and leaves	Reddish yellow	1.50 ± 0.38

Values are presented as mean ± SD from three independent extractions.

**Table 2:** Retention index (RI) and relative peak area (%) of compounds in EOs extracted from 13 species of *Lamiaceae*.

=Relative Content (%)															
Compounds ^i^	RI Exp ^ii^	RI Lit	*R.of * ^iii^	*S.ba*	*N.ca*	*M.ca*	*P.fr*	*E.ci*	*O.vu*	*S.sc*	*P.mo*	*S.te*	*T.ci*	*T.vu*	*T.mo*
Limonene	1018	1020 ^a^	-- ^iv^	41.78 ^a^ ± 1.11	1.3 ^h^ ± 0.43	10.57 ^g^ ± 0.91	24.86 ^d^ ± 1.3	15.56 ^f^ ± 0.33	34.48 ^b^ ± 1.27	29.65 ^c^ ± 0.98	22.35 ^e^ ± 0.89	24.2 ^d^ ± 0.98	16.67 ^f^ ± 0.11	--	--
Citronellol	1208	1207 ^a^	--	--	--	--	0.56 ^d^ ± 0.06	7.32 ^a^ ± 0.05	2.27 ^b^ ± 0.11	1.15 ^c^ ± 0.13	1.02 ^c^ ± 0.84	--	--	--	--
β-Linalool	1081	1082 ^a^	0.93 ^h^ ± 0.03	8.94 ^f^ ± 0.27	--	0.71 ^h^ ± 0.02	17.28 ^c^ ± 0.32	3.9 ^g^ ± 0.79	11.77 ^e^ ± 0.81	13.81 ^d^ ± 0.24	0.75 ^h^ ± 0.09	4.74 ^g^ ± 1.50	20.61 ^b^ ± 0.94	23.64 ^a^ ± 0.98	35.00 ^a^ ± 2.79
β-Terpineol	1120	1120 ^a^	0.69 ^c^ ± 0.13	--	0.01 ^d^ ± 0.00	--	0.72 ^c^ ± 0.03	2.52 ^a^ ± 0.20	--	--	--	1.64 ^b^ ± 0.40	--	--	--
β-Ocimene	1034	1036 ^a^	--	--	0.01 ^c^ ± 0.00	--	1.03 ^b^ ± 0.03	1.21 ^b^ ± 0.18	1.72 ^a^ ± 0.26	1.75 ^a^ ± 0.19	--	0.92 ^b^ ± 0.69	--	--	--
Geraniol	1249	1250 ^a^	1.06 ^c^ ± 0.06	--	0.02 ^d^ ± 0	--	0.69 ^c^ ± 0.13	--	2.66 ^b^ ± 0.1	8.41 ^a^ ± 1.15	1.13 ^c^ ± 0.29	1.25 ^c^ ± 0.35	--	--	--
Nerol	1342	1342 ^a^	--	--	--	1.28 ^b^ ± 0.67	--	3.49 ^a^ ± 1.16	--	--	--	--	--	--	--
(Z)-Nerolidol	1545		--	--	--	--	0.23 ^c^ ± 0.02	0.04 ^c^ ± 0.01	--	1.35 ^b^ ± 0.13	--	2.69 ^a^ ± 1.45	0.23 ^c^ ± 0.03	--	--
Bornylene	1508		0.47 ^d^ ± 0.01	--	--	0.23 ^ef^ ± 0.01	0.01 ^h^ ± 0	0.05 ^gh^ ± 0.01	--	0.25 ^e^ ± 0.06	0.14 ^fg^ ± 0.01	--	4.44 ^b^ ± 0.07	13.26 ^a^ ± 0.15	1.36 ^c^ ± 0.12
β-Terpinrol	1125	1126 [18]	--	--	--	--	--	--	--	--	--	--	3.56 ^b^ ± 0.14	4.97 ^a^ ± 0.37	--
Pulegone	1230	1236 ^a^	--	0.27 ^c^ ± 0.01	41.32 ^a^ ± 2.76	1.12 ^c^ ± 0.02	--	--	--	--	--	40 ^b^ ± 0.15	--	--	--
Menth-8-en-1-ol	1151	1144 [19]	--	--	--	0.98 ^c^ ± 0.1	1.14 ^b^ ± 0.19	--	--	--	--	--	0.52 ^d^ ± 0.06	2.05 ^a^ ± 0.02	--
Menthone	1150	1155 ^a^	--	0.48 ^c^ ± 0.19	11.3 ^a^ ± 1.41	1.23 ^b^ ± 0.09	--	--	--	--	--	--	--	--	--
Eucalyptol	1023	1020 ^a^	38.00 ^a^ ± 0.52	--	--	--	--	--	--	11.35 ^b^ ± 10.14	--	--	--	--	7.31 ^b^ ± 0.42
(+)-4-Carene	1018	998 ^a^	3.16 ^a^ ± 0.20	0.16 ^c^ ± 0.03	0.02 ^d^ ± 0.00	--	0.14 ^c^ ± 0.02	0.07 ^cd^ ± 0.02	0.35 ^b^ ± 0.08	--	0.07 ^cd^ ± 0.01	0.06 ^cd^ ± 0.03	--	0.07 ^cd^ ± 0.02	0.07 ^cd^ ± 0.01
Verbenone	1191	1204 ^a^	1.68 ^b^ ± 0.01	0.20 ^d^ ± 0.03	--	4.06 ^a^ ± 0.06	--	--	--	0.21 ^d^ ± 0.01	0.33 ^c^ ± 0.06	--	--	--	--
Caryophyllene	1424	1421 ^a^	1.61 ^d^ ± 0.05	11.19 ^a^ ± 0.1	5.14 ^b^ ± 0.27	1.37 ^e^ ± 0.15	--	--	--	--	1.58 ^de^ ± 0.24	--	--	--	4.20 ^c^ ± 0.2
cis-p-Menthone	1166	1172 ^a^	1.6 ^f^ ± 0.09	4.56 ^c^ ± 0.3	--	11.16 ^a^ ± 1.68	--	--	--	--	--	7 ^b^ ± 0.43	--	--	--
Camphene	943	958 ^a^	1.59 ^a^ ± 0.13	--	--	--	0.07 ^c^ ± 0.01	--	--	--	0.16 ^b^ ± 0.01	--	--	--	--
β-Pinene	970	972 ^a^	1.32 ^a^ ± 0.09	0.2 ^c^ ± 0.03	0.01 ^c^ ± 0	0.71 ^b^ ± 0.06	--	--	--	--	--	--	0.05 ^c^ ± 0.01	--	1.24 ^a^ ± 0.46
Acetic acid	1277	--	1.28 ^a^ ± 0.16	--	0.01 ^c^ ± 0.01	--	--	--	--	--	--	--	--	--	0.84 ^b^ ± 0.05
β-Phellandrene	1030	1023 [18]	0.95 ^a^ ± 0.01	0.13 ^b^ ± 0.01	0.01 ^ef^ ± 0.01	0.07 ^cd^ ± 0.01	--	0.05 ^cde^ ± 0.01	--	0.03 ^ef^ ± 0.01	0.14 ^b^ ± 0.02	0.09 ^c^ ± 0.08	0.05 ^de^ ± 0.01	--	--
(+)-2-Bornanone	1138	--	0.82 ^b^ ± 0.01	0.27 ^c^ ± 0.03	--	--	--	--	--	--	2.74 ^a^ ± 0.09	--	--	--	--
γ-Terpinene	1161	1162 ^a^	0.57 ^a^ ± 0.13	0.26 ^bc^ ± 0.11	--	0.1 ^cde^ ± 0.05	0.01 ^e^ ± 0.00	0.05 ^de^ ± 0.02	0.02 ^e^ ± 0.00	0.21 ^bcd^ ± 0.01	--	0.36 ^b^ ± 0.33	--	--	--
L-Menthol	1150.4	1143.95 ^a^	--	--	--	34.83 ^a^ ± 1.27	--	--	--	--	0.14 ^b^ ± 0.02	--	--	--	--
p-Menthone	1159	1157 ^a^	--	0.53 ^b^ ± 0.20	--	6.93 ^a^ ± 0.71	--	--	--	--	--	--	--	--	--
Isopulegone	1140	1150 ^a^	--	10.94 ^a^ ± 9.42	3.01 ^b^ ± 0.16	--	--	--	1.26 ^b^ ± 0.18	--	--	--	0.16 ^b^ ± 0.05	0.98 ^b^ ± 0.53	--
cis-2-Pinanol	1182	1180 ^a^	--	--	--	3.75 ^a^ ± 0.29	--	--	--	--	0.14 ^b^ ± 0.20	--	--	--	--
3-Octanol	985	981 ^a^	--	--	--	1.58 ^a^ ± 0.12	--	0.01 ^b^ ± 0.001	--	--	--	--	--	--	--
o-Cymene	1025.4	1051.3	--	--	0.01 ^c^ ± 0.00	1.37 ^a^ ± 0.15	0.02 ^c^ ± 0.01	0.03 ^c^ ± 0.01	0.08 ^bc^ ± 0.01	0.09 ^bc^ ± 0.01	--	0.19 ^b^ ± 0.10	--	--	--
α-Terpinene	1240	1029 ^a^	0.47 ^b^ ± 0.003	0.14 ^c^ ± 0.04	--	0.80 ^a^ ± 0.08	0.02 ^ef^ ± 0.01	0.04 ^ef^ ± 0.01	0.02 ^ef^ ± 0.002	0.04 ^ef^ ± 0.003	--	0.06 ^ed^ ± 0.04	0.05 ^efd^ ± 0.01	0.07 ^de^ ± 0.03	0.10 ^cd^ ± 0.01
O-Eugenol	1350	1362 ^a^	--	0.77 ^b^ ± 0.03	--	--	--	--	--	--	1.11 ^a^ ± 0.78	--	--	--	0.67 ^b^ ± 0.08
δ-Cadinene	1514	1537 ^a^	--	2.23 ^c^ ± 0.11	--	0.21 ^d^ ± 0.04	--	--	4.16 ^b^ ± 0.34	--	4.82 ^b^ ± 0.86	--	--	7.31 ^a^ ± 1.07	--
α-Muurolene	1440	1440 [20]	--	--	--	0.59 ^b^ ± 0.3	--	--	--	--	31.93 ^a^ ± 2.99	--	1.43 ^b^ ± 0.16	--	--
Cedr-8-ene	1405	1414 ^a^	--	1.87 ^a^ ± 0.04	--	0.14 ^c^ ± 0.01	--	--	--	--	0.97 ^b^ ± 0.05	--	--	--	--
Citral	1240.5	1240.5 ^a^	--	--	--	--	--	0.36 ^b^ ± 0.33	0.24 ^b^ ± 0.04	0.06 ^c^ ± 0.01	1.86 ^a^ ± 0.20	0.02 ^c^ ± 0.01	--	--	--
Copaene	1397	1376 ^a^	--	0.63 ^a^ ± 0.11	--	0.21 ^b^ ± 0.01	--	--	--	--	--	--	--	--	0.15 ^c^ ± 0.02
Humulene	1456	1454 ^a^	--	0.55 ^a^ ± 0.05	--	--	--	--	--	--	0.37 ^b^ ± 0.03	--	--	--	--
Borneol	1148	1148 ^a^	--	--	0.01 ^c^ ± 0.01	--	--	--	--	0.11 ^bc^ ± 0.02	0.13 ^bc^ ± 0.01	0.21 ^b^ ± 0.20	--	--	7.91 ^a^ ± 0.25
(+)-car-3-ene	995	1000 ^a^	--	--	--	0.34 ^c^ ± 0.12	--	0.53 ^b^ ± 0.19	--	--	--	--	--	--	1.72 ^a^ ± 0.18
4-Carvomenthenol	1161	1162 ^a^	0.25 ^b^ ± 0.13	--	--	--	--	--	--	--	--	--	--	--	0.80 ^a^ ± 0.03
Geradiene	990	976	--	--	--	--	0.01 ^d^ ± 0.002	5.15 ^a^ ± 0.93	1.51 ^b^ ± 0.01	1.27 ^b^ ± 0.11	--	0.04 ^d^ ± 0.02	0.67 ^c^ ± 0.07	--	--
Anethole	1264	1269 ^a^	--	--	--	--	--	--	--	--	--	7.16 ^a^ ± 4.21	--	0.02 ^b^ ± 0.003	--
1-Octen-3-ol	1065	1080	--	--	1.45 ^a^ ± 0.47	0.44 ^c^ ± 0.16	1.19 ^b^ ± 0.18	--	--	--	--	--	--	--	--
Anisaldehyde	1227	1200 [18]	--	--	--	--	--	--	--	--	--	5.33 ^a^ ± 2.09	--	--	0.52 ^b^ ± 0.05
Thymol	1266	1266 ^a^	--	--	--	--	--	--	--	--	--	--	11.00 ^c^ ± 1.12	13.3 ^b^ ± 1.46	25.00 ^a^ ± 0.72
Perilla aldehyde	1246	1241 ^a^	--	--	0.95 ^b^ ± 0.24	--	35.00 ^a^ ± 2.68	1.46 ^b^ ± 0.62	--	--	--	--	--	--	--
Carveol	1254	1261 ^a^	--	--	1.12 ^b^ ± 0.55	2.13 ^a^ ± 0.27	--	--	--	--	--	--	--	--	--
Carvacrol	1278	1275 [21]	--	--	--	--	--	--	28.00 ^a^ ± 0.36	--	--	--	--	--	5.00 ^b^ ± 0.5
α-Pinene	931	930 ^a^	16.00 ^a^ ± 1.77	0.26 ^b^ ± 0.11	0.78 ^b^ ± 0.17	0.07 ^b^ ± 0.04	--	--	--	--	0.33 ^b^ ± 0.1	0.36 ^b^ ± 0.04	--	--	--
Nepetalactone	1365	1393 ^a^	--	--	16.30 ^b^ ± 2.41	--	--	35.00 ^a^ ± 0.79	--	--	--	--	--	--	--
Total	--	--	72.45	86.36	82.78	86.97	82.98	76.84	88.54	69.74	72.21	96.32	59.44	65.67	91.89

Values within a row followed by different letters are significantly different (*p* < 0.05, Duncan’ s Multiple Range Test). ^a^ RI lit values were obtained from the NIST Chemistry WebBook, NIST Standard Reference Database Number 69 (https://doi.org/10.18434/T4D303, accessed on 18 September 2026). ^i^ Compound are listed in order of elution from a methyl silicone capillary column. ^ii^ Retention index (RI) relative ton-alkanes (C6–C40) on the same methyl silicone capillary column. ^iii^ Abbreviations: *R.of*: *Rosmarinus officinalis*; *S.ba*: *Scutellaria baicalensis*; *N.ca*: *Nepeta cataria*; *M.ca*: *Mentha canadensis*; *P.fr: Perilla frutescens*; *E.ci: Elsholtzia ciliata*; *O.vu: Origanum vulgare*; *S.sc: Scutellaria scordifolia*; *P.mo: Phlomoides mongolica*; *S.te*: *Schizonepeta tenuifolia*; *T.ci: Thymus citriodorus*; *T.vu: Thymus vulgaris*; *T.mo: Thymus mongolicus*. ^iv^ Undetected.

**Table 3:** Inhibition zone diameter (mm) of EOs extracted from 13 *Lamiaceae* species.

EOs	Gram-Negative			Gram-Positive	
	*E. coli*	*K. Pneumoniae*	*S. marcescens*	*S. aureus*	*S. haemolyticus*
*Rosmarinus officinalis*	10.50 ± 0.20 ^d^	9.47 ± 0.45 ^e^	NI	NI	NI
*Scutellaria baicalensis*	7.50 ± 0.17 ^f^	NI	7.13 ± 0.02 ^h^	6.60 ± 0.10 ^f^	NI
*Nepeta cataria*	NI	7.27 ± 0.25 ^g^	8.10 ± 0.36 ^f^	7.36 ± 0.28 ^ef^	NI
*Mentha canadensis*	NI	7.27 ± 0.25 ^g^	8.10 ± 0.36 ^f^	7.37 ± 0.29 ^ef^	NI
*Perilla frutescens*	9.89 ± 0.60 ^d^	9.90 ± 0.17 ^e^	10.90 ± 0.20 ^d^	30.17 ± 1.29 ^a^	15.23 ± 0.97 ^d^
*Elsholtzia ciliata*	8.17 ± 0.15 ^f^	9.62 ± 0.71 ^e^	9.43 ± 0.40 ^e^	15.80 ± 0.63 ^c^	12.27 ± 0.92 ^e^
*Origanum vulgare*	22.04 ± 0.83 ^ab^	18.11 ± 0.53 ^a^	15.17 ± 0.06 ^a^	30.83 ± 2.45 ^a^	34.50 ± 1.35 ^b^
*Scutellaria scordifolia*	8.34 ± 0.20 ^ef^	7.23 ± 0.21 ^g^	7.53 ± 0.21 ^gh^	9.07 ± 0.15 ^de^	NI
*Phlomis mongolica*	9.50 ± 0.27 ^de^	8.67 ± 0.36 ^f^	9.27 ± 0.15 ^e^	10.57 ± 0.29 ^d^	9.47 ± 0.55 ^f^
*Schizonepeta tenuifolia*	10.01 ± 0.56 ^d^	9.73 ± 0.40 ^w^	7.80 ± 0.17 ^fg^	10.57 ± 0.35 ^d^	9.47 ± 0.06 ^f^
*Thymus citriodorus*	22.76 ± 0.77 ^ab^	12.99 ± 0.30 ^c^	12.43 ± 0.33 ^c^	21.02 ± 1.18 ^b^	35.37 ± 1.86 ^b^
*Thymus vulgaris*	19.73 ± 1.58 ^c^	10.97 ± 0.12 ^d^	12.12 ± 0.50 ^c^	15.69 ± 0.99 ^c^	23.83 ± 1.43 ^c^
*Thymus mongolicus*	23.43 ± 1.12 ^a^	14.40 ± 0.35 ^b^	13.16 ± 0.39 ^b^	22.39 ± 2.03 ^b^	38.13 ± 1.95 ^a^

Values are presented as mean ± SD from three independent experiments. Values within the same column followed by different letters are significantly different (*p* < 0.05, Duncan’s multiple range test). NI, no inhibition; NI indicates that no inhibition zone was observed beyond the 6-mm paper disc.

**Table 4:** MIC values (μg/mL) of selected EOs against opportunistic pathogens.

EOs	*E. coli*	*K. pneumoniae*	*S. marcescens*	*S. aureus*	*S. haemolyticus*
*Origanum vulgare*	2	2	4	2	2
*Thymus citriodorus*	2	8	8	2	2
*Thymus vulgaris*	4	8	8	4	8
*Thymus mongolicus*	2	4	4	2	2

Gentamicin was used as the positive control. The MIC values for the bacterial strains listed in Table 4 were 0.5, 0.5, 1, 0.25, and 0.5 μg/mL, respectively. MIC values were determined in three independent experiments.

**Table 5:** Cytotoxicity of EOs extracted from 13 *Lamiaceae* species.

EOs	IC_50_ (μg/mL)	
	LNCaP	HepG2
*Rosmarinus officinalis*	0.51 ± 0.04 ^bcde^	0.81 ± 0.08 ^a^
*Scutellaria baicalensis*	0.59 ± 0.08 ^bc^	0.34 ± 0.05 ^d^
*Nepeta cataria*	0.57 ± 0.25 ^bcd^	0.56 ± 0.08 ^bc^
*Mentha canadensis*	0.44 ± 0.03 ^cde^	0.59 ± 0.12 ^bc^
*Perilla frutescens*	0.40 ± 0.04 ^cde^	0.35 ± 0.07 ^d^
*Elsholtzia ciliata*	0.36 ± 0.08 ^de^	0.31 ± 0.09 ^d^
*Origanum vulgare*	0.53 ± 0.12 ^bcde^	0.83 ± 0.07 ^a^
*Scutellaria scordifolia*	0.66 ± 0.08 ^b^	0.55 ± 0.12 ^bc^
*Phlomis mongolica*	0.67 ± 0.14 ^b^	0.65 ± 0.08 ^b^
*Schizonepeta tenuifolia*	0.68 ± 0.09 ^b^	0.44 ± 0.05 ^cd^
*Thymus citriodorus*	0.60 ± 0.12 ^bc^	0.45 ± 0.1 ^cd^
*Thymus vulgaris*	0.35 ± 0.06 ^e^	0.55 ± 0.15 ^bc^
*Thymus mongolicus*	0.95 ± 0.15 ^a^	0.64 ± 0.08 ^b^

Values are presented as mean ± SD from three independent experiments. Different letters in the same column indicate significant differences (*p* < 0.05).

## Data Availability

The relevant data supporting this study’s findings are available on reasonable request from the corresponding author.

## Supplementary Materials

The following supporting information can be downloaded at: https://www.mdpi.com/article/10.3390/plants15203084/s1, Figure S1: Photographs of essential oils extracted from 13 *Lamiaceae* species. (A) *R.of*; (B) *S.ba*; (C) *N.ca*; (D) *M.ca*; (E) *P.fr*; (F) *E.ci*; (G) *O.vu*; (H) *S. sc*; (I) *P.mo*; (J) *S.te*; (K) *T.mo*; (L) *T.vu*; (M) *T.ci*. Figure S2: TIC of the 13 *Lamiaceae* EOs by GC-MS. (A) *R.of*; (B) *S.ba*; (C) *N.ca*; (D) *M.ca*; (E) *P.fr*; (F) *E.ci*; (G) *O.vu*; (H) *S.sc*; (I) *P.mo*; (J) *S.te*; (K) *T.mo*; (L) *T.vu*; (M) *T.ci*. Figure S3: Selectivity index (SI) of 13 *Lamiaceae* EOs against LNCaP (A) and HepG2 (B) cells. Data are presented as mean ± SD from three independent experiments. Figure S4: Western blot analysis of inflammatory proteins in skin tissue. Expression analysis (A, B) and semi-quantitative analysis (C, D) of proteins (COX-2, TNF-α, NF-κB, IKK, Akt and PKC) after treatment with the control group, model group and 9 kinds of EOs. Data are presented as mean ± SD (n = 6 mice per group). Figure S5: qPCR analysis of the expression of inflammation-related genes in skin tissue. Relative mRNA levels of *COX-2*, *TNF-α*, *NF-κB*, *IKK*, *Akt* and *PKC* in the control group, the model group and after treatment with 9 kinds of EOs. Data are presented as mean ± SD (n = 6 mice per group). Figure S6: Pathological analysis (H&E staining) and immunohistochemical analysis (COX-2, TNF-α) of skin tissues after treatment with 9 kinds of EOs. Representative images are shown; six mice were analyzed per group (n = 6). Table S1: Major compounds with a relative content exceeding 5% in each type of essential oil. Table S2: The antioxidant activity of 13 *Lamiaceae* EOs in terms of scavenging DPPH free radicals. Table S3: Cytotoxicity of 13 *Lamiaceae* EOs against normal cell lines. Table S4: *R*^2^ of the dose–response curves for the 13 *Lamiaceae* EOs against LNCaP, HepG2, RWPE-1, and THLE-2 cells. Table S5: Complete Spearman correlation matrix between major volatile compounds and bioactivity indices. Table S6: Primers for the six anti-inflammatory genes.

## Abbreviations

The following abbreviations are used in this manuscript:

Abbreviation	Full Form
Akt	Protein Kinase B
CCK-8	Cell Counting Kit-8
COX-2	Cyclooxygenase-2
DPPH	2,2-Diphenyl-1-picrylhydrazyl
EO	Essential Oil
FDR	False Discovery Rate
GC-MS	Gas Chromatography-Mass Spectrometry
IKK	IκB Kinase
MIC	Minimum Inhibitory Concentration
NF-κB	Nuclear Factor-kappa B
HCA	Hierarchical clustering analysis
PCA	Principal Component Analysis
PKC	Protein Kinase C
qPCR	Quantitative Polymerase Chain Reaction
qRT-PCR	Quantitative Real-Time Polymerase Chain Reaction
SDS	Sodium Dodecyl Sulfate
SI	Selectivity Index
TIC	Total Ion Chromatogram
TNF-α	Tumor Necrosis Factor-alpha
