# Supplementary material for: Comparative Analysis of GC-MS Chemical Profiles and Multidimensional Biological Activities of Essential Oils from Thirteen Lamiaceae Plants
Source: Plants (Basel). 2026 Oct 9;15(20):3084. doi: 10.3390/plants15203084 (PMC13708472; doi:10.3390/plants15203084)
Supplement: Supplementary file 1 [file plants-15-03084-s001.zip › plants-4539849-supplementary.pdf]

Supporting Information

Comparative Analysis of GC-MS Chemical Profiles and Multidimensional Biological Activities of Essential Oils from Thirteen *Lamiaceae* Plants

Feng Han[1, 2], Yingmei Ma[3], Yuting Bai[4], Shuangyi Zhang[4], Yan Zhao[1, 2,*], Xiujie Yin[5,*]

1. College of Grassland Science, Inner Mongolia Agricultural University, Hohhot, Inner Mongolia, 010011, China.

2. Key Laboratory of National Forestry and Grassland Administration on Native Grass Breeding, College of Grassland Science, Inner Mongolia Agricultural University, Hohhot 010018, China.

3. College of Desert Management, Inner Mongolia Agricultural University, Huhehot, Inner Mongolia, 010018, China.

4. College of Veterinary Medicine, Inner Mongolia Agricultural University, Hohhot City, Inner Mongolia, 010011, China.

5. College of Animal Science and Technology, Northeast Agricultural University, Harbin, Heilongjiang, 150030, China.

*Corresponding authors:

E-mail addresses: Zhaoyannmg@imau.edu.cn;

yinxiujie@126.com.

Figure S1 Photographs of essential oils extracted from 13 *Lamiaceae* species. (A) *R.of*; (B) *S.ba*; (C) *N.ca*; (D) *M.ca*; (E) *P.fr*; (F) *E.ci*; (G) *O.vu*; (H) *S. sc*; (I) *P.mo*; (J) *S.te*; (K) *T.mo*; (L) *T.vu*; (M) *T.ci*.

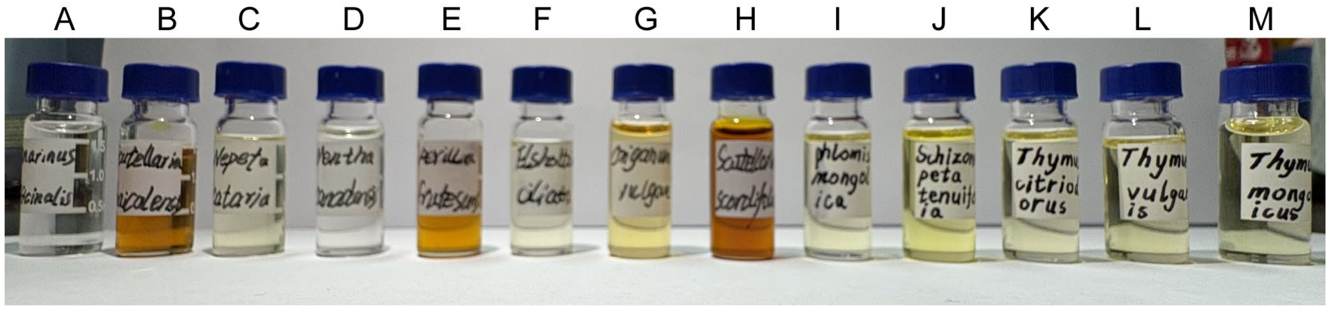

Abbreviations: *R.of: Rosmarinus officinalis; S.ba: Scutellaria baicalensis; N.ca: Nepeta cataria; M.ca: Mentha canadensis; P.fr: Perilla frutescens; E.ci: Elsholtzia ciliata; O.vu: Origanum vulgare; S.sc: Scutellaria scordifolia; P.mo: Phlomoides mongolica; S.te: Schizonepeta tenuifolia; T.ci: Thymus citriodorus; T.vu: Thymus vulgaris; T.mo: Thymus mongolicus.*

Figure S2 TIC of the 13 *Lamiaceae* EOs by GC-MS. (A) *R.of*; (B) *S.ba*; (C) *N.ca*; (D) *M.ca*; (E) *P.fr*; (F) *E.ci*; (G) *O.vu*; (H) *S.sc*; (I) *P.mo*; (J) *S.te*; (K) *T.mo*; (L) *T.vu*; (M) *T.ci*.

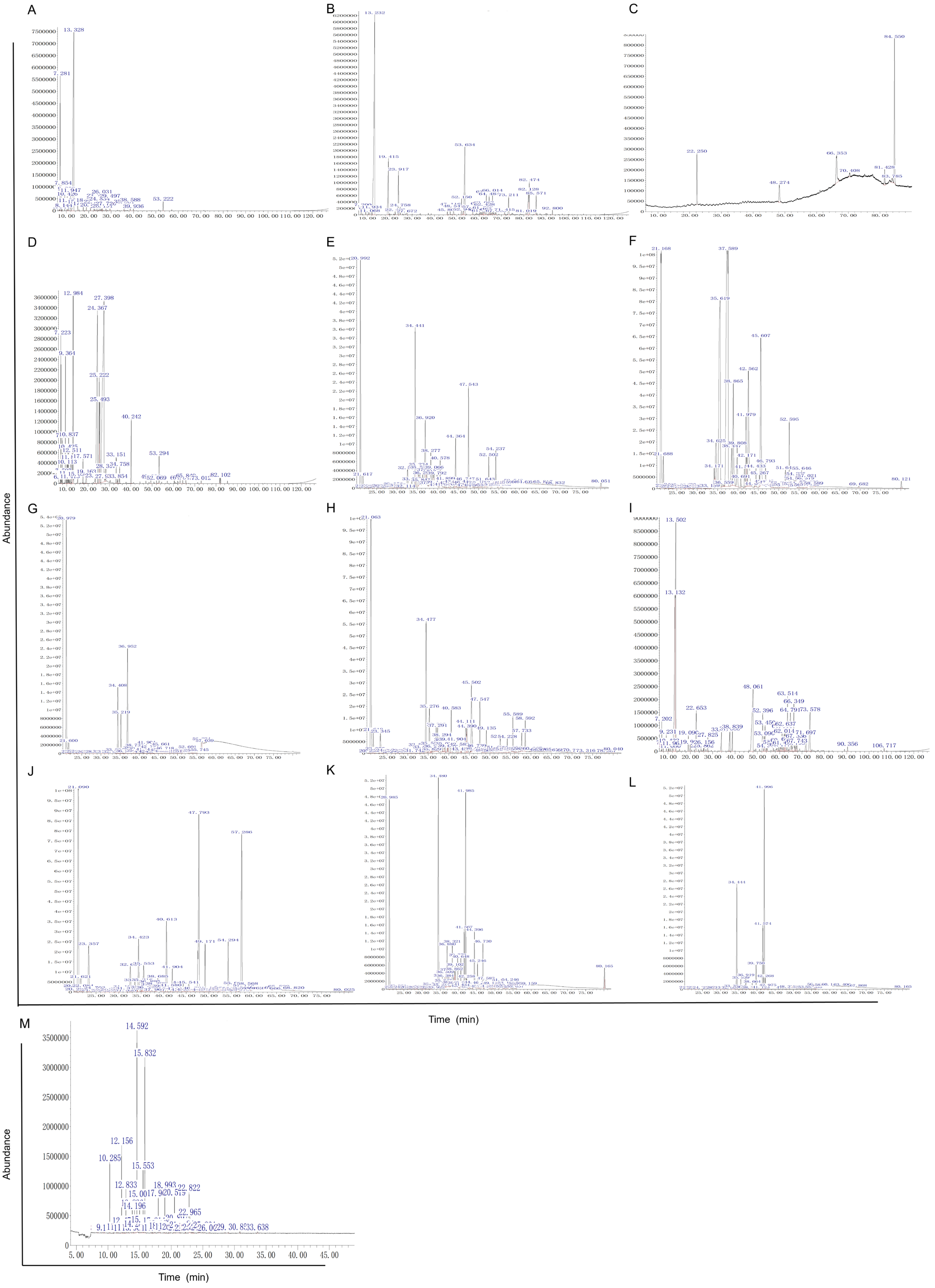

Figure S3 Selectivity index (SI) of 13 *Lamiaceae* EOs against LNCaP (A) and HepG2 (B) cells. Data are presented as mean ± SD from three independent experiments.

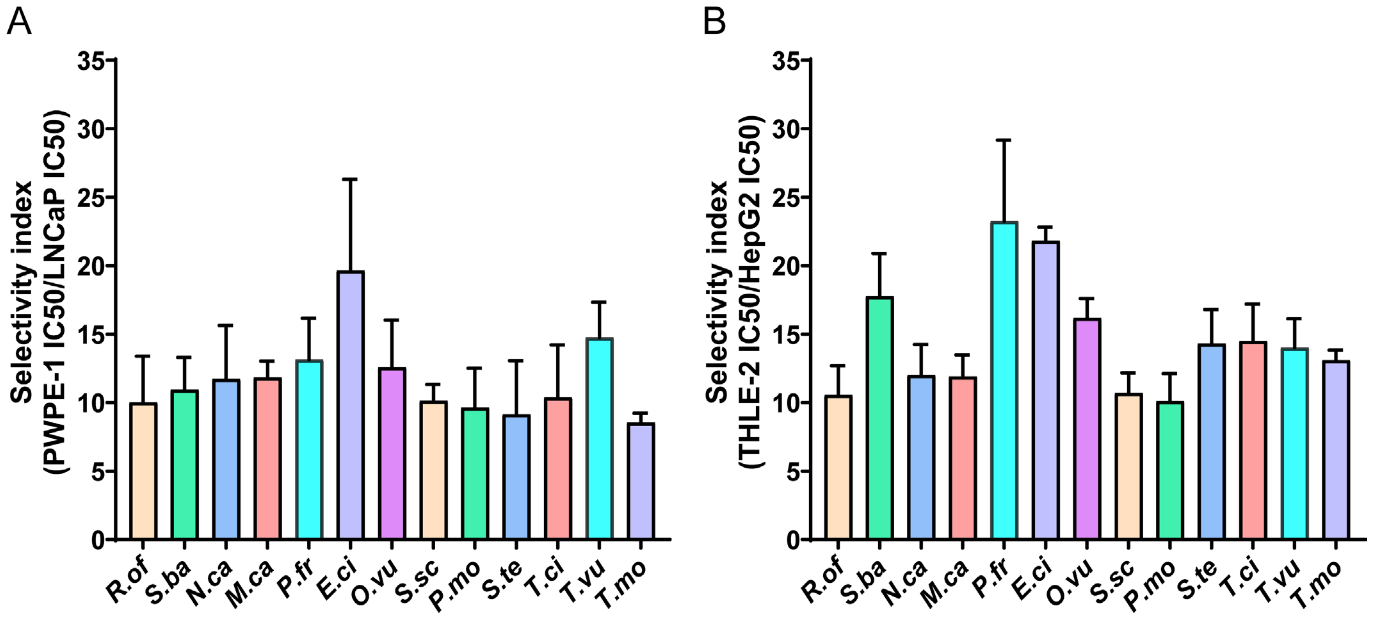

Figure S4 Western blot analysis of inflammatory proteins in skin tissue. Expression analysis (A, B) and semi-quantitative analysis (C, D) of proteins (COX-2, TNF-α, NF-κB, IKK, Akt and PKC) after treatment with the control group, model group and 9 kinds of EOs. Data are presented as mean ± SD (n = 6 mice per group).

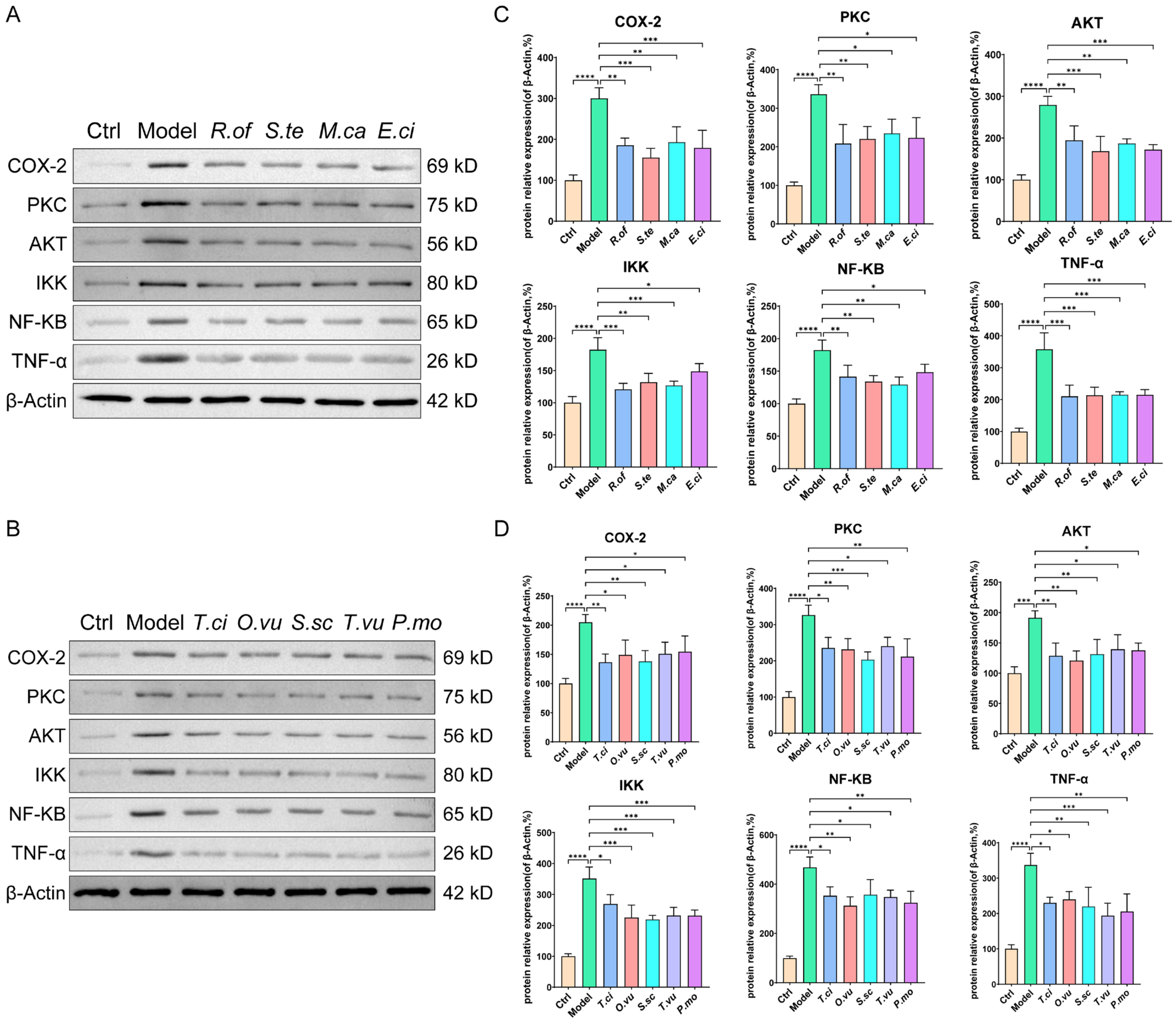

Figure S5 qPCR analysis of the expression of inflammation-related genes in skin tissue. Relative mRNA levels of *COX-2*, *TNF-α*, *NF-κB*, *IKK*, *Akt* and *PKC* in the control group, the model group and after treatment with 9 kinds of EOs. Data are presented as mean ± SD (n = 6 mice per group).

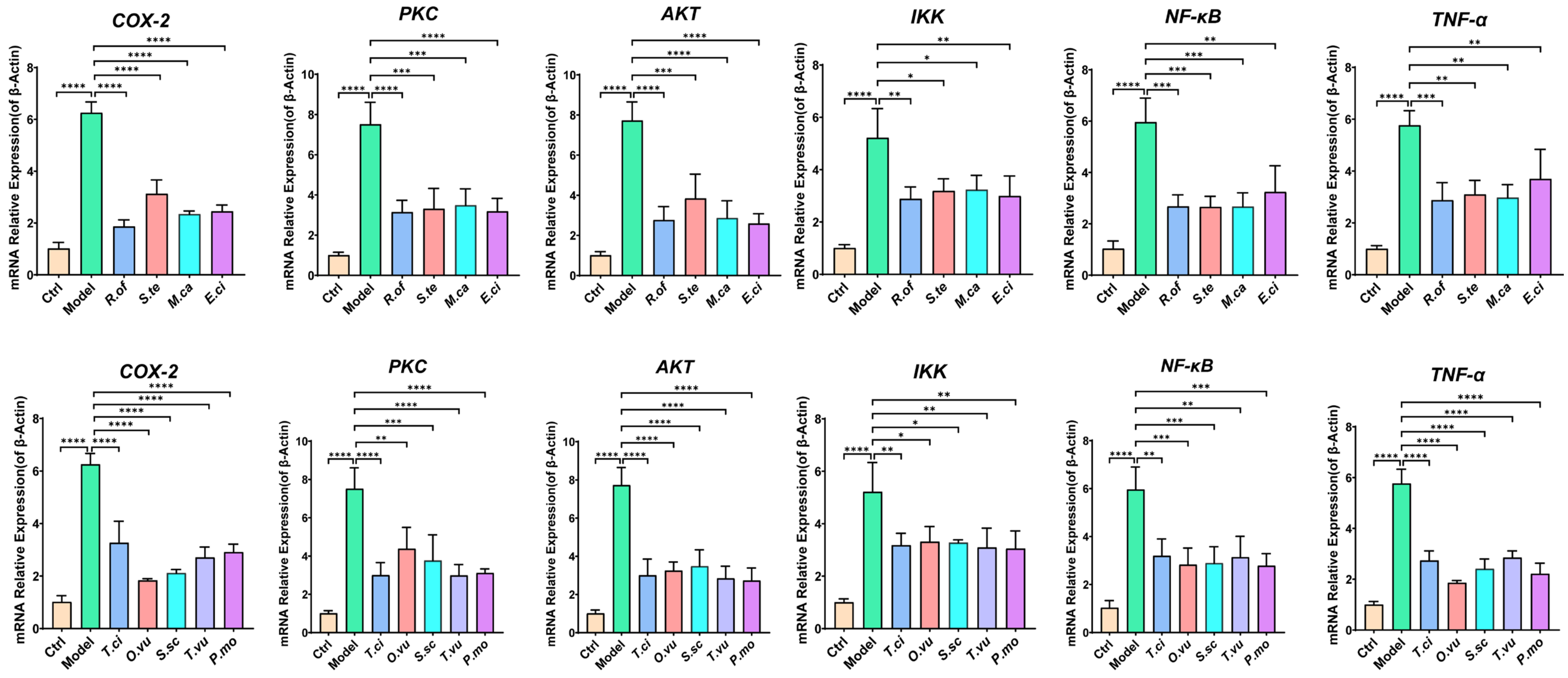

Figure S6 Pathological analysis (H&E staining) and immunohistochemical analysis (COX-2, TNF-α) of skin tissues after treatment with 9 kinds of EOs. Representative images are shown; six mice were analyzed per group (n = 6).

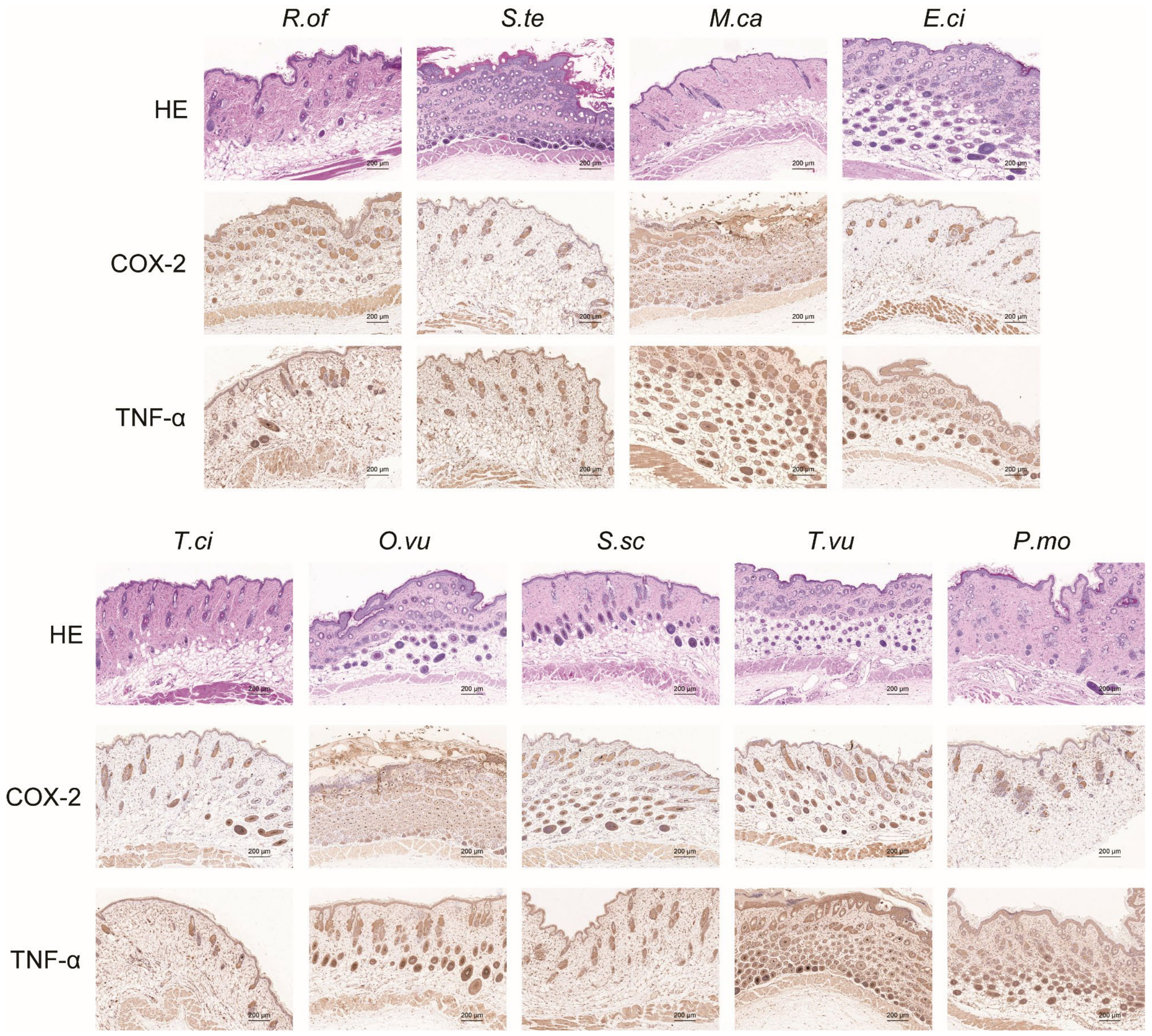

Table S1 Major compounds with a relative content exceeding 5% in each type of essential oil.

Latin name	Major compounds	Relative content (%)
Rosmarinus officinalis	Eucalyptol	38.00 ± 0.52
	α-Pinene	16.00 ± 1.77
Scutellaria baicalensis	Limonene	41.78 ± 1.11
	Caryophyllene	11.19 ± 0.1
	Isopulegone	10.94 ± 9.42
	β-Linalool	8.94 ± 0.27
Nepeta cataria	Pulegone	41.32 ± 2.76
	Nepetalactone	16.30 ± 2.41
	Menthone	11.30 ± 1.41
	Caryophyllene	5.14 ± 0.27
Mentha canadensis	L-Menthol	34.83 ± 1.27
	cis-p-Menthone	11.16 ± 1.68
	Limonene	10.57 ± 0.91
	p-Menthone	6.93 ± 0.71
Perilla frutescens	Perilla aldehyde	35.00 ± 2.68
	Limonene	24.86 ± 1.3
	β-Linalool	17.28 ± 0.32
Elsholtzia ciliata	Nepetalactone	35.00 ± 0.79
	Limonene	15.56 ± 0.33
	Citronellol	7.32 ± 0.05
	Geradiene	5.15 ± 0.93
Origanum vulgare	Limonene	34.48 ± 1.27
	carvacrol	28.00 ± 0.36
	β-Linalool	11.77 ± 0.81
Scutellaria scordifolia	Limonene	29.65 ± 0.98
	β-Linalool	13.81 ± 0.24
	Eucalyptol	11.35 ± 10.14
	Geraniol	8.41 ± 1.15
Phlomis mongolica	α-Muurolene	31.93 ± 2.99
	Limonene	22.35 ± 0.89
Schizonepeta tenuifolia	Pulegone	40.00 ± 0.15
	Limonene	24.20 ± 0.98
	Anethole	7.16 ± 4.21
	Anisaldehyde	5.33 ± 2.09
Thymus citriodorus	β-Linalool	20.61 ± 0.94
	Limonene	16.67 ± 0.11
	thymol	11.00 ± 1.12
Thymus vulgaris	β-Linalool	23.64 ± 0.98
	thymol	13.3 ± 1.46
	bornylene	13.26 ± 0.15
	δ-Cadinene	7.31 ± 1.07
Thymus mongolicus	β-Linalool	35.00 ± 2.79
	thymol	25.00 ± 0.72
	Borneol	7.91 ± 0.25
	Eucalyptol	7.31 ± 0.42

Values represent the mean relative content (%) from three technical injections of one representative EO sample for each species.

Table S2 The antioxidant activity of 13 *Lamiaceae* EOs in terms of scavenging DPPH free radicals.

EOs	DPPH（%）
Rosmarinus officinalis	55.56 ± 12.63[a]
Scutellaria baicalensis	50.93 ± 14.76
Nepeta cataria	27.78 ± 10.39
Mentha canadensis	48.15 ± 10.72
Perilla frutescens	51.85 ± 9.17
Elsholtzia ciliata	31.48 ± 13.67
Origanum vulgare	26.85 ± 11.64
Scutellaria scordifolia	25.93 ± 11.19
Phlomis mongolica	28.70 ± 7.97
Schizonepeta tenuifolia	34.26 ± 10.23
Thymus citriodorus	45.37 ± 13.29
Thymus vulgaris	44.44 ± 8.18
Thymus mongolicus	48.15 ± 12.9

Values are expressed as mean ± SD from three independent experiments.

Table S3 Cytotoxicity of 13 *Lamiaceae* EOs against normal cell lines.

EOs	IC_{50} (μg/mL)	
	RWPE-1	THLE-2
Rosmarinus officinalis	5.11±1.71	8.56±1.27
Scutellaria baicalensis	6.46±1.40	6.04±1.06
Nepeta cataria	6.69±2.22	6.73±1.25
Mentha canadensis	5.21±0.53	7.03±0.94
Perilla frutescens	5.26±1.20	8.14±2.07
Elsholtzia ciliata	7.07±0.08	6.76±0.31
Origanum vulgare	6.67±1.83	13.43±1.19
Scutellaria scordifolia	6.69±0.79	5.89±0.81
Phlomis mongolica	6.46±1.93	6.57±1.31
Schizonepeta tenuifolia	6.22±2.66	6.29±1.10
Thymus citriodorus	6.22±2.30	6.52±1.12
Thymus vulgaris	5.17±0.90	7.71±1.16
Thymus mongolicus	8.12±0.66	7.20±0.41

Values are expressed as mean ± SD from three independent experiments.

Table S4 R^2 of the dose–response curves for the 13 *Lamiaceae* EOs against LNCaP, HepG2, RWPE-1, and THLE-2 cells.

EO	LNCaP			HepG2			RWPE-1			THLE-2		
	R^2-1	R^2-2	R^2-1	R^2-2	R^2-3	R^2-1	R^2-2	R^2-3	R^2-3	R^2-1	R^2-2	R^2-3
Rosmarinus officinalis	0.855	0.958	0.958	0.802	0.995	0.941	0.974	0.961	0.933	0.966	0.977	0.976
Scutellaria baicalensis	0.982	0.990	0.829	0.948	0.924	0.925	0.955	0.971	0.978	0.994	0.998	0.990
Nepeta cataria	0.952	0.970	0.938	0.911	0.973	0.889	0.990	0.956	0.993	0.987	0.966	0.980
Mentha canadensis	0.995	0.987	0.932	0.930	0.917	0.936	0.997	0.968	0.966	0.960	0.982	0.970
Perilla frutescens	0.995	0.984	0.980	0.951	0.995	0.979	0.948	0.969	0.984	0.997	0.997	0.988
Elsholtzia ciliata	0.981	0.977	0.794	0.931	0.992	0.964	0.991	0.878	0.996	0.992	0.993	0.979
Origanum vulgare	0.952	0.977	0.981	0.773	0.910	0.894	0.985	0.938	0.981	0.981	0.985	0.965
Scutellaria scordifolia	0.990	0.985	0.942	0.863	0.987	0.946	0.938	0.754	0.986	0.973	0.996	0.993
Phlomis mongolica	0.981	0.976	0.900	0.926	0.826	0.868	0.960	0.985	0.957	0.986	0.983	0.991
Schizonepeta tenuifolia	0.948	0.935	0.911	0.968	0.954	0.933	0.955	0.972	0.978	0.993	0.978	0.986
Thymus citriodorus	0.971	0.978	0.967	0.969	0.963	0.971	0.976	0.891	0.979	0.986	0.994	0.979
Thymus vulgaris	0.992	0.988	0.872	0.988	0.980	0.921	0.948	0.999	0.984	0.996	0.984	0.995
Thymus mongolicus	0.977	0.921	0.924	0.991	0.979	0.928	0.991	0.927	0.892	0.990	0.973	0.981

Data are from three independent experiments fitted by a four-parameter logistic regression model.

Table S5 Complete Spearman correlation matrix between major volatile compounds and bioactivity indices.

Major volatile compounds	DPPH(1/IC_{50})	*E. coli*	*K. Pneumoniae*	*S. marcescens*	*S. aureus*	*S. haemolyticus*	LNCaP(1/IC_{50})	HepG2(1/IC_{50})
carvacrol	-0.096/0.919	0.560/0.046*	0.630/0.020*	0.630/0.020*	0.577/0.023*	0.576/0.033*	-0.253/0.555	-0.525/0.132
thymol	0.272/0.511	0.685/0.018*	0.588/0.041*	0.588/0.041*	0.394/0.213	0.704/0.010*	-0.126/0.783	-0.061/0.881
perillaldehyde	0.095/0.876	-0.370/0.370	-0.032/0.944	0.099/0.831	0.212/0.610	-0.004/0.984	0.464/0.212	0.473/0.199
β-linalool	0.234/0.599	0.740/0.010*	0.614/0.041*	0.553/0.069	0.625/0.035*	0.733/0.011*	-0.093/0.840	0.173/0.680
limonene	-0.355/0.381	-0.196/0.662	-0.208/0.625	-0.108/0.818	0.147/0.767	-0.122/0.799	-0.199/0.671	0.302/0.486
eucalyptol	0.153/0.753	0.260/0.549	-0.093/0.853	-0.324/0.456	-0.268/0.543	-0.176/0.708	-0.279/0.520	-0.378/0.372
pulegone	-0.050/0.933	-0.605/0.048*	-0.484/0.159	-0.450/0.208	-0.528/0.133	-0.550/0.116	-0.148/0.770	0.175/0.723
nepetalactone	-0.307/0.505	-0.472/0.239	-0.184/0.728	-0.018/0.977	-0.087/0.855	-0.153/0.765	0.306/0.497	0.315/0.477
L-menthol	-0.031/0.968	-0.389/0.372	-0.319/0.479	-0.092/0.856	-0.210/0.645	-0.279/0.557	-0.026/0.688	-0.332/0.456
α-pinene	0.069/0.896	-0.401/0.321	-0.514/00.169	-0.706/0.019	-0.775/0.007*	-0.680/0.027*	-0.203/0.636	-0.233/0.576

Values are presented as Spearman's correlation coefficient (ρ) / Benjamini–Hochberg FDR-adjusted q value. * indicates $q < 0.05$. All correlation analyses were based on 13 EO samples (n = 13).

Table S6 Primers for the six anti-inflammatory genes.

Gene	Forward	Reverse
COX-2	TCAGCATTCATTCCTCTACATA	ACAACACTCACATATTCATACATT
PKC	GCACAATGACAGAGAAGA	GGAAGCAGCAATAGAGTT
AKT	CAGTTTGAGACCACACAT	GCGTCAGTCCTTAATAGTT
NF-KB	TTCAAGTGCCTTAATAGC	AAGAGCAAGAGTCCAATA
IKK	GCACTCAGAAGCAATAGA	AGATGGAACCAGAACTTG
TNF-α	AGTCTGTATCCTTCTAAC	TTCTGAGTAGTTGTTGAA
